# Association of the retail food environment, BMI, dietary patterns, and socioeconomic position in urban areas of Mexico

**DOI:** 10.1371/journal.pgph.0001069

**Published:** 2023-02-23

**Authors:** Elisa Pineda, Diana Barbosa Cunha, Mansour Taghavi Azar Sharabiani, Christopher Millett

**Affiliations:** 1 Centre for Health Economics & Policy Innovation (CHEPI), Imperial College Business School, South Kensington Campus, London, United Kingdom; 2 Policy Evaluation Unit, School of Public Health, Imperial College London, London, United Kingdom; 3 Social Medicine Institute, State University of Rio de Janeiro, Rio de Janeiro, Brazil; 4 NOVA National School of Public Health, Public Health Research Centre, Comprehensive Health Research Center, CHRC, NOVA University Lisbon, Lisbon, Portugal; PLOS: Public Library of Science, UNITED STATES

## Abstract

The retail food environment is a key modifiable driver of food choice and the risk of non-communicable diseases (NCDs). This study aimed to assess the relationship between the density of food retailers, body mass index (BMI), dietary patterns, and socioeconomic position in Mexico. Cross-sectional dietary data, BMI and socioeconomic characteristics of adult participants came from the nationally representative 2012 National Health and Nutrition Survey in Mexico. Geographical and food outlet data were obtained from official statistics. Densities of food outlets per census tract area (CTA) were calculated. Dietary patterns were determined using exploratory factor analysis and principal component analysis. The association of food environment variables, socioeconomic position, BMI, and dietary patterns was assessed using two-level multilevel linear regression models. Three dietary patterns were identified—the healthy, the unhealthy and the carbohydrates-and-drinks dietary pattern. Lower availability of fruit and vegetable stores was associated with an unhealthier dietary pattern whilst a higher restaurant density was associated with a carbohydrates-and-drinks pattern. A graded and inverse association was observed for fruit and vegetable store density and socioeconomic position (SEP)—lower-income populations had a reduced availability of fruit and vegetable stores, compared with higher-income populations. A higher density of convenience stores was associated with a higher BMI when adjusting for unhealthy dietary patterns. Upper-income households were more likely to consume healthy dietary patterns and middle-upper-income households were less likely to consume unhealthy dietary patterns when exposed to high densities of fruit and vegetable stores. When exposed to a high concentration of convenience stores, lower and upper-lower-income households were more likely to consume unhealthy dietary patterns. Food environment and sociodemographic conditions within neighbourhoods may affect dietary behaviours. Food environment interventions and policies which improve access to healthy foods and restrict access to unhealthy foods may facilitate healthier diets and contribute to the prevention of NCDs.

## Introduction

Food choice is influenced by a complex set of determinants that include biological, economic, and social factors as well as attitudes, knowledge and beliefs [1, 2]. Unhealthy food choices are a major modifiable risk factor for diet related non-communicable diseases (NCDs) [3]. The retail food environment has been identified as a key driver of food choice [4, 5]. It encompasses the accessibility of food retailers, the availability of healthy or unhealthy foods and beverages within these establishments, and their affordability and promotion [6]. Unhealthy retail food environments have been associated with a higher risk of diet related NCDs [7].

The implementation of the North American Free Trade Agreement (NAFTA) agreement between Mexico, the US and Canada in 1994 allowed an increased importation of ultra-processed foods and growth in internationally owned fast-food chain retailers along with the export of fruits and vegetables to the US [8, 9]. Mexico has experienced a nutritional transition, including a decline in fruit and vegetable intake, and an increasing trend of overweight and obesity [10, 11], such that the country now has one of the highest prevalence of obesity worldwide at 75% [12]. With the intention to curb the obesity trend, in 2014, Mexico became one of the first countries to implement a sugar-sweetened beverage (SSB) tax [13]. Since then, several policies have targeted nutritional labelling and televised food marketing. However, the burden of obesity continues to grow along with an increasingly obesogenic food environment which offers a wide range of high-calorie and low-nutrient foods and beverages at low prices [12, 14, 15].

Previous studies have shown that healthier dietary intakes can be enabled through supportive food environments which facilitate access to healthy and affordable food choices, such as fruits and vegetables [16]. Inequalities in access to affordable, healthy, and nutritious food can contribute to health disparities. Research has shown that low-income areas have limited access to healthy foods which may limit the ability of individuals to have healthier diets, exposing them to a greater risk of obesity and NCDs [16].

The price of fruits and vegetables has been rising more than most other foods, including energy-dense processed foods in Mexico [17]. This is concerning and reflects a current lack of policies to address the declining consumption of fruit and vegetables in the country. In addition, the consumption of energy dense foods high in saturated fat, salt and sugar (HFSS) and ultra-processed foods, has increased [18]. Mexico has become one of the highest consumers of HFSS and SSBs in Latin America [19]. From 2009 to 2014, sales of ultra-processed foods increased by 5% Mexico and SSBs contributed to 22% of the total energy intake per capita [19]. Main policy recommendations include to reduce consumption of ultra-processed foods. Yet, although previous research has assessed the relationship of the food environment and obesity in Mexico [20, 21], evidence of the impact of the retail food environment on dietary intake and particularly dietary patterns is limited in Mexico. Furthermore, no actions have been undertaken at national level to improve the food environment to enable healthier food choices. Therefore, the aims of this study were 1) to test the association of dietary patterns and the retail food environment, 2) to test the association of the retail food environment and BMI whilst considering dietary patterns and 3) to test the role of socioeconomic position (SEP) in dietary patterns and the retail food environment.

This study builds on previous research in which the association of body mass index (BMI) and food environment in Mexico were studied [20]. In addition to BMI the present study considers the interaction of dietary patterns with the food environment and the confounding effect of diet when testing the association of the food environment and BMI. We hypothesized that a healthy dietary pattern would be associated with a higher supermarket and fruit and vegetable store availability due to the potential higher availability of healthy foods (e.g. fruits and vegetables) and that this association would be more evident in upper-income SEP households due to previous studies showing greater availability of healthy foods and outlets in higher-income neighbourhoods [22–24]. We also hypothesized that unhealthy dietary patterns would be associated with food stores which focused their sales on unhealthy low-nutrient, energy-dense foods.

## Methods

### Ethics statement

Ethical approval was sought and obtained by the National Institute of Health (INSP) in Mexico from the NIH Research Ethics Committee to carry out the National Health and Nutrition Survey (ENSANUT).

### Study design

This study involved a secondary analysis of cross-sectional and population-based survey data. Dietary and sociodemographic data were obtained from the 2012 Mexican National Survey of Health and Nutrition (ENSANUT, acronym in Spanish) [25]. This was a national probabilistic survey with state level representation by urban and rural strata and an oversampling of households with the greatest deprivation levels in the country. The sample for ENSANUT included the overrepresentation of households in the country in conditions of greater vulnerability, on the assumption that the support of health and social programs is focused on these households. All used survey data was previously anonymised by the National Institute of Health and Nutrition (INSP, acronym in Spanish) in Mexico.

### Data collection

#### Anthropometric and sociodemographic data

Body weight and height were measured by trained personnel for the 2012 ENSANUT survey [26]. Sociodemographic data, including sex, age, car ownership, type of health service user, participation in food programmes (type of programme is described in the results section) and region were extracted for this study from the 2012 ENSANUT survey [26] and captured in a database in STATA 14 [27].

Physical activity was self-reported and was assessed by the ENSANUT survey through the International physical activity questionnaire (IPAQ short), which was previously validated [26]. Physical activity levels were defined as active, moderately active, and inactive/sedentary according to the criteria stablished by the World Health Organization [26, 28].

Household socioeconomic position (SEP) was obtained from the 2012 ENSANUT [12] which considered demographic and socioeconomic characteristics, including characteristics of the head of the family, sociodemographic structure, characteristics of the home, household goods, family consumption patterns and characteristics of the geographical area of residence based on the 2010 National Income and Expenditure Survey [29]. Distribution of SEP household characteristics was described by predicted decile. Deciles were then compared with a measure of poverty to create quintiles which were then equally assigned to each household member. A lower quintile indicates lower-income whilst a higher quintile indicates a higher-income [20, 29]. Study population data focused on the general adult population and excluded data from women who were pregnant, survey participants <18 years of age, and participants without a valid, measured weight and height. Participants with BMI values of >3 standard deviations from the mean were excluded (<15 kg/m2 and >58 kg/m2) in case of possible underlying illnesses, eating disorders or implausible values.

#### Dietary intake

Dietary information was obtained through a validated, semi quantitative food frequency questionnaire (FFQ) [26, 30], which was applied to 11% of the ENSANUT participant sample population [26]. The analytical sample size was N = 1,572. The FFQ included data regarding the consumption of 140-food items. For each food item, portion size and frequency per day, week and year was registered [26].

#### Geocoding of individuals and food outlets

The geographical areas of study were urban neighbourhoods in the country of Mexico. Census tract areas (CTA) were used as a proxy for neighbourhoods. A CTA in Mexico is defined as a geographic area formed by of a set of blocks delimited by streets or identifiable pathways with land used for residential industrial or commercial services [31]. Urban CTAs contain a population of ≥2,500 inhabitants. There were 55,427 urban CTAs in this study, with mean area of 0.59 km^2^. The smallest CTA was 0.009 km^2^ and the largest 5.20 km^2^ [31].

Anonymised participants from the 2012 ENSANUT were geocoded to the centroid of their urban CTA with ArcGIS 10.2.2 (ESRI, Redlands, CA). Exact ENSANUT participant’s geolocation (home address) was unavailable to protect participant’s privacy. Geographic coordinates of food outlets were obtained from INEGI, 2014 [32]. On-site verification of nine geographic area samples was undertaken to verify the geolocation, existence, and type of food outlet. Urban CTAs were grouped into a single shapefile, which was spatially merged with geolocation and sociodemographic characteristics of participants and food outlets.

#### Food retail data

Retail data were obtained from the 2014 Economic Census from the National Institute of Statistics and Geography (INEGI, acronym in Spanish) [31]. Food outlets encompassed convenience stores fast-food outlets, restaurants, supermarkets and fruit and vegetable stores. Classification of food outlets was according to INEGI and revised on our previous study [20]. To summarise, for this study, food outlets, including informal and mobile food carts, which specialised in pizzas, hamburgers, hotdogs, and fried chicken were classified as fast-food outlets. Outlets that mainly sold SSBs and unhealthy snacks were classified as convenience stores. We assumed that all convenience stores and fast-food outlets sold mainly SSBs, snacks and ultra-processed foods. Food outlets with an á la carte menu, that included healthy food alternatives with sitting options available, were classified as restaurants. Mega-supermarkets and grocery stores, which offered greater food options than convenience stores, including fruits and vegetables, were classified as supermarkets. Fruit and vegetable stores outlets included informal fruit stands, small shops, and farmer market style locations. These establishments were characterised by mainly selling fruit and/or vegetables.

Store data, which included type of store and location, was obtained from the 2014 economic census from the National Institute of Statistics and Geography (INEGI) [31]. Density of food outlets was calculated considering the total number of food outlet (e.g. restaurants, fast-food outlets, convenience stores and fruit and vegetable stores) per CTA. Density units are expressed as food outlet per CTA km^2^. The density of food outlets by state was mapped to visualize the distribution in the country. Due to a lower availability of food outlets, a higher availability of informal commerce not in record and a higher density of food crops) [20, 21] rural areas were excluded from this study.

### Statistical analyses

#### Dietary patterns

The 140-food items from the FFQs were aggregated grouping together food items according to their nutritional and common habitual dietary consumption (i.e., how foods are usually paired up for consumption in the study population (e.g., tortillas with beans or milk with cereal), based on previous research [33, 34]. Dietary patterns were computed using exploratory factor analysis (FA), principal component as the extraction method and varimax rotation. Kaiser-Meyer-Olkin test was undertaken to investigate the adequacy of FA to the data. A scree plot was used to select the number of factors to retain (S1 Fig). Food items with factor loading ≥0.30 were retained in the pattern. Factor scores were computed and included in the regression analysis using SAS OnDemand for Academics (SAS Institute Inc., Cary, USA) [35].

#### Association of dietary patterns and the retail food environment

The main outcome of this study was dietary intake represented as dietary patterns and the exposure was food outlet store density (food outlet count per census tract area). Statistical models were constructed after drawing the postulated relationship of variables through directed acyclic graphs (DAG) [36], which captured the dependence structure of multiple variables and allow more robust conclusions about the direction of association. Three multilevel linear regression models were used to test the association between food outlet density and dietary patterns. State, or CTA were used as a second random effect in the three models (Table 1).

**Table 1:** Multilevel linear regression models used to test the association of food outlet density and dietary patterns.

Model	Covariates	Second random effect level
**Model A**	Age, sex, and SEP.	State
**Model B**	Age, sex, SEP, physical activity level, car ownership, neighbourhood deprivation level.	CTA
**Model C**	Age, sex, SEP, CTA deprivation level, and urbanity level.	State

SEP: socioeconomic position, quintiles: lowest, second lowest, middle, second highest and highest.

Physical activity was self-reported and was assessed in the ENSANUT survey through the International physical activity questionnaire (IPAQ short), which was previously validated [26]. Physical activity levels were defined as active, moderately active, and inactive/sedentary according to the criteria stablished by the World Health Organization [26, 28].

CTA: Census tract area. CTA deprivation level: defined as low or high.

Urbanity level: defined as metropolitan area (i.e., ≥1 million inhabitants), urban centres (i.e., cities with ≥15,000 and <1 million inhabitants), or rural (excluded from this study) [37].

#### Association of the retail food environment and BMI whilst considering dietary patterns

Models A, B and C were replicated to test first the association of the food environment and BMI considering the interaction of the food environment with the dietary patterns and second, to test the association of the food environment and BMI whilst considering the dietary patterns as confounders. To assess sex differences, results were also stratified by sex.

#### Role of SEP in dietary patterns and the retail food environment

To assess the influence of household SEP, Model A, B and C were considered and stratified by household SEP to understand if there were variations within the population. Also, the interaction between socioeconomic position and food retail density was tested. In addition, two-level multinomial logistic regressions with random effects were undertaken to assess the role of socioeconomic aspects of the environment and their influence on dietary patterns in urban CTAs of Mexico.

Multicollinearity was measured for each model by considering variance inflation factors. Variance inflation factors did not exceed the value of 4.0 for any of the included variables and were therefore all included in the models. Survey design and weights were accounted in all models and statistical analyses were undertaken in STATA 14 [27].

## Results

### General characteristics of the population and the retail food environment

From the study sample, (N  =  5,080), 56% were female, 35% (n = 2,824) had a sedentary lifestyle, 12% (n = 585) owned a car and 14% (n = 690) participated in a form of food programme (Table 2). Food programmes encompassed the Oportunidades programme (n = 395, 68%), a national conditional cash transfer program targeting poor and extremely poor households; Oportunidades school scholarships (n = 22, 4%); funding support for the elderly (n = 8, 1%); Progresa medical support (n = 21, 4%); monetary support from the Ayuda programme; milk supply programmes Liconsa or Conasupo (n = 46, 8%); food pantries from DIF (n = 24, 4%); food pantries from other organisations, social kitchens and canteens (n = 11, 2%); school breakfasts (n = 1, 0.2%); other educational scholarships (n = 13, 2%); non-governmental or civil organization (n = 1, 0.2%); other financial support for the elderly (n = 1, 0.2%); other (n = 38, 6%).

**Table 2:** Sociodemographic, and economic characteristics of Mexican adults aged 18+ inhabiting urban areas of Mexico ^a^.

Variable	Total N (%)
**Gender**	
** Men**	2,256 (44)
** Women**	2,824 (56)
**Age**	
** 18–24**	640 (13)
** 25–34**	506 (10)
** 35–44**	628 (12)
** 45–54**	451 (9)
** 55–64**	336 (7)
** 65+**	323 (6)
** Missing**	2,196 (43)
**Physical activity**	
** Active**	401 (8)
** Moderately active**	266 (5)
** Inactive**	1,793 (35)
** Missing**	2,620 (52)
Household SEP^b^	
** Highest**	1,027 (22)
** Second highest**	960 (21)
** Middle**	938 (20)
** Second lowest**	955 (20)
** Lowest**	803 (17)
** Missing**	397 (8)
**Car ownership**	
** Owns a car**	585 (12)
** Does not own a car**	2,295 (45)
** Missing**	2,200 (43)
**Region**	
** South**	1,729 (34)
** North**	1,242 (24)
** Centre**	1,769 (35)
** Metropolitan area**	340 (7)
**Area deprivation level**	
** Low**	3,041 (60)
** High**	2,039 (40)
**Urbanicity**	
** Rural (excluded)**	1,830 (36)
** Urban**	1,025 (20)
** Metropolitan**	2,225 (44)
**Food programme**	
** Participated**	581 (11)
** Did not participate**	2,303 (45)
** Did not respond/did not know**	2,196 (43)
**Health service**	
**Covered**	663 (13)
**Not covered**	2,221 (44)
**Did not respond/did not know**	2,196 (43)

^a^ Provided data is from the nationally representative, cross-sectional, 2012 ENSANUT (National Health and Nutrition) Survey in Mexico.

^b^ Physical activity levels were defined as active, moderately active, and inactive/sedentary according to the criteria stablished by the World Health Organization [26, 28].

^c^ SEP: socioeconomic position, quintiles: lowest, second lowest, middle, second highest and highest.

‘Missing’ indicates number of participants that did not have a record due to not responding the survey for this section or not knowing what to respond.

N  =  5,080

The assessment of the food environment in Mexico was undertaken as part of the first phase of this study and has been published and is described elsewhere [20]. To summarise, out of 72,892 CTAs in Mexico, only 10,145 CTAs (14%) had access to a fruit and vegetable store. When looking at the distribution of food outlets in Mexico, a higher fruit and vegetable store concentration was evident in the centre and South of Mexico compared with the North and metropolitan regions of Mexico. The North of Mexico shows a very low availability of fruit and vegetable stores compared to the rest of the country. In addition, convenience stores and fast-food outlets were widely available throughout the country.

#### Dietary patterns

Kaiser’s measure of sampling adequacy was 0.79 which indicated that FA was appropriate method due to a correlation of variables [38]. Three dietary patterns were retained, according to the scree plot (S1 Fig), which together explained a variance of 26.3% for factor 1; 26.1% for factor 2; and 16.4% for factor 3 of the total variance of the data. We retained the factors above the inflection point of the curve of the scree plot (patterns that showed and eigen value >1/0).

For each of the food groups, a factor loading value ≥0.28 indicated that the food group was included in the factor. Therefore, considering all food groups and corresponding factor loadings, factor 1 was classified as a healthy pattern, characterized by a higher consumption, in comparison of other factors, of fruits and vegetables, cooked meals, pulses, fish and seafood, meat, fermented dairy, soups, bread, and natural drinks. Factor 2 was classified as an unhealthy dietary pattern, mainly composed of fats, high meat and fatty meals, sugar and desserts, sausages, dressings, soda, fast-food, ready to eat soups, potato chips and candy. Factor 3 was denominated as carbohydrates-and-drinks pattern, which encompassed juice and natural drinks, whole wheat products, milk, and refined cereal (S1 Table).

#### Associations of dietary patterns and the retail food environment

Fruit and vegetable store density was inversely associated with unhealthy dietary patterns. This finding was replicated in the three statistical models that were tested (Models A, B and C) (Table 2). Restaurants were repeatedly and positively associated with the carbohydrates-and-drinks type of dietary pattern for models A (β: 0.003 95% CI: 0.0016, 0.005, P < 0.001), B (β: 0.004, 95%CI: 0.002, 0.005, P < 0.001) and C (β: 0.003 95%CI: 0.001, 0.005, P < 0.001) and inversely associated with the unhealthy dietary pattern for models B and C. Fast-food outlets, supermarkets and convenience stores were not statistically significantly associated with any type of dietary pattern (Table 3).

**Table 3:** Multivariate associations between dietary patterns and food outlet density; data from the nationally representative, cross-sectional, 2012 ENSANUT^a^—Dietary assessment component.

Food outlet density (number of stores/CTA)	Factor 1 Healthy pattern		Factor 2 Unhealthy pattern		Factor 3 Carbohydrate & drinks pattern	
	β (95% CI)	P-value	β (95% CI)	P-value	β (95% CI)	P-value
Fruit and vegetable stores Model A	0.002 (-0.0001, 0.005)	0.064	-0.002 (-0.004, -0.0002)	0.029	0.001 (-0.001, 0.003)	0.435
Fruit and vegetable stores Model B	0.002 (-0.0006, 0.004)	0.131	-0.003 (-0.005, -0.001)	<0.001	0.002 (-0.0006, 0.004)	0.129
Fruit and vegetable stores Model C	0.002 (-0.0001, 0.005)	0.060	-0.002 (-0.004, -0.0003)	0.025	0.001(-0.001, 0.003)	0.397
Fast-food outlets Model A	0.001 (-0.005, 0.008)	0.692	-0.004 (-0.01, 0.001)	0.127	0.006 (-0.007, 0.014)	0.077
Fast-food outlets Model B	0.001 (-0.006, 0.009)	0.734	-0.004 (-0.01, -0.36)	0.094	0.007 (-0.0001, 0.014)	0.053
Fast-food outlets Model C	0.001 (-0.0062, 0.008)	0.781	-0.005 (-0.011, 0.0003)	0.066	0.005 (-0.002, 0.012)	0.141
Restaurants- Model A	0.0008 (-0.0008, 0.002)	0.344	-0.001 (-0.002, 0.00005)	0.060	0.003 (0.0016, 0.005)	<0.001
Restaurant Model B	0.0006 (-0.001, 0.002)	0.482	-0.002 (-0.004, -0.001)	<0.001	0.004 (0.002, 0.005)	<0.001
Restaurant Model C	0.0007 (-0.0009, 0.002)	0.404	-0.001 (-0.003, -0.0002	0.027	0.003 (0.001, 0.005)	<0.001
Supermarkets Model A	0.014 (0.024, 0.053)	0.478	-0.03 (-0.057, 0.004)	0.094	0.012 (-0.027, 0.052)	0.541
Supermarkets Model B	0.01 (-0.031, 0.05)	0.635	-0.028 (-0.060, -0.003)	0.081	0.020 (-0.020, 0.060)	0.323
Supermarkets Model C	0.014 (-0.025, 0.053)	0.479	-0.028 (-0.059, 0.003)	0.077	0.012 (-0.27, 0.522)	0.533
Convenience stores Model A	0.0002 (-0.001, 0.001)	0.797	-0.0007 (-0.002, 0.0003)	0.163	-0.0007 (-0.002, 0.0007)	0.327
Convenience stores Model B	0.0002 (-0.001, 0.001)	0.792	0.005 (-0.003, 0.12)	0.239	0.000007 (-0.001, 0.001)	0.991
Convenience stores Model C	0.0002 (-0.001, 0.001)	0.708	-0.0009 (-0.002, 0.0002)	0.112	-0.0005 (-0.002, 0.0009)	0.480

Model A: Age, sex, and socioeconomic position, N = 1,572

Model B: Model A + socioeconomic position, physical activity, car ownership, neighbourhood deprivation level, CTA (2nd level), N = 1,568

Model C: Model A + deprivation and urbanity of CTA, N = 1,572

### Association of the retail food environment and BMI whilst considering dietary patterns

When testing the association of the food environment and dietary patterns, a higher density of supermarkets and fruit and vegetable stores was inversely associated with the carbohydrates-and-drinks dietary pattern (factor 3) for model A (β_supermarkets_ = -0.36, 95%CI: -0.65, -0.06, P = 0.02); (β_fruit and vegetable stores_ = -0.40, 95%CI: -0.69, -0.11, P = 0.01); model B (β_supermarkets_ = -0.33, 95%CI: -0.63, 0.03, P = 0.03); (β_fruit and vegetable stores_ -0.37, 95%CI: -0.67, -0.08, P = 0.01); and model C (β_supermarkets_ = -0.35, 95%CI: -0.65, -0.06, P = 0.02); (β_fruit and vegetable stores_ = -0.40, 95%CI: -0.69, -0.10, P = 0.01) (S2 Table).

When testing the association of the food environment and BMI whilst considering dietary patterns as a confounder, a higher density of convenience stores showed a statistically significant association with a higher risk of obesity (β_convenience stores_ = 0.06, 95%CI: 0.01, 0.12, P = 0.03) (S3 Table). No statistically significant findings were identified when stratifying results by sex (S4 Table).

### Role of SEP in dietary patterns and the retail food environment

When assessing the relationship of fruit and vegetable store density and household SEP, a graded and inverse association was observed in which lower-income households had a reduced availability of fruit and vegetable stores, compared with higher-income households (β: -0.007, 95% CI -0.0011, -0.004), P < 0.001) (Table 4). Furthermore, when stratifying the association of dietary patterns with fruit and vegetable store density by household SEP, upper-income households were more likely to consume healthy dietary patterns (β: 0.004, 95% CI: 0.0004, 0.007, P = 0.027). Additionally, middle-upper-income households were less likely to consume unhealthy dietary patterns when exposed to high densities of fruit and vegetable stores (β: -0.003, 95% CI: -0.006, -0.0002, P *=* 0.036) (Table 5).

**Table 4:** Association of household SEP and food outlet density; data from the nationally representative, cross-sectional, 2012 ENSANUT^a^, N = 22,219.

SEP	Food outlet density ^a^	
	β (95% CI)	P-value
**Fruit and vegetable store**		
** Upper-income**	Ref	
** Middle-upper-income**	-0.0009 (-0.002, 0.0005)	0.185
** Middle-income**	-0.002 (-0.004, -0.0007)	0.006
** Upper-lower-income**	-0.005 (-0.007, -0.002)	<0.001
** Lower-income**	-0.007 (-0.011, -0.004)	<0.001
**Fast-food outlets**		
** Upper-income**	Ref	
** Middle-upper-income**	0.002 (-0.004, 0.008)	0.503
** Middle-income**	-0.0005 (-0.011, 0.001)	0.152
** Upper-lower-income**	-0.026 (-0.034, -0.018)	<0.001
** Lower-income**	-0.061 (-0.072, -0.050)	<0.001
**Restaurants**		
** Upper-income**	Ref	
** Middle-upper-income**	-0.002 (-0.004, -0.001)	<0.001
** Middle-income**	-0.004 (-0.005, -0.002)	<0.001
** Upper-lower-income**	-0.007 (-0.009, -0.006)	<0.001
** Lower-income**	-0.014 (-0.017, -0.012)	<0.001
**Supermarkets**		
** Upper-income**	Ref	
** Middle-upper-income**	-0.048 (-0.078, -0.018)	0.001
** Middle-income**	-0.086 (-0.119, -0.054)	<0.001
** Upper-lower-income**	-0.161 (-0.202, 0.119)	<0.001
** Lower-income**	-0.198 (0.253, -0.144)	<0.001
**Convenience stores**		
** Upper-income**	Ref	
** Middle-upper-income**	0.006 (0.005, 0.007)	<0.001
** Middle-income**	0.006 (0.005, 0.008)	<0.001
** Upper-lower-income**	0.006 (0.004, 0.007)	<0.001
** Lower-income**	0.005 (0.003, 0.006)	<0.001

SEP: socioeconomic position, quintiles: lowest, second lowest, middle, second highest and highest.

Results indicate β coefficients and 95% confidence intervals from two-level multinomial logistic regressions with random effects were undertaken to assess the role of socioeconomic aspects of the environment and their influence on dietary patterns in urban CTAs of Mexico.

^a^ Model A, adjusted by age and sex.

**Table 5:** Association of dietary pattern and food outlet density, stratified by household SEP.

SEP	Healthy dietary pattern (Factor 1) ^a^		Unhealthy dietary pattern (Factor 2)^a^		Carbohydrate dietary pattern (Factor 3)^a^	
	β (95%CI)	P-value	β (95%CI)	P-value	β (95%CI)	P-value
**Fruit and vegetable stores**						
** Upper-income**	0.004 (0.0004, 0.007)	0.027	-0.003 (-0.006, 0.0005)	0.105	-0.001 (-0.006, 0.003)	0.582
** Middle-upper-income**	0.001 (-0.002, 0.005)	0.524	-0.003 (-0.006, -0.0002)	0.036	0.002 (-0.002, 0.006)	0.296
** Middle-income**	0.003 (-0.009, 0.02)	0.581	0.002 (-0.007, 0.01)	0.582	0.004 (-0.007, 0.015)	0.501
** Upper-lower-income**	-0.001 (-0.007, 0.004)	0.634	-0.005 (-0.01, 0.00001)	0.051	0.004 (-0.001, 0.01)	0.159
** Lower-income**	0.01 (-0.019, 0.045)	0.434	0.01 (-0.01, 0.03)	0.352	-0.004 (-0.03, 0.02)	0.752
**Fast-food outlets**						
** Upper-income**	0.005 (-0.007, 0.017))	0.443	-0.007 (-0.018, 0.004)	0.209	-0.001 (-0.017, 0.013)	0.846
** Middle-upper-income**	0.001 (-0.010, 0.012)	0.822	-0.009 (-0.019, -0.0003)	0.043	0.013 (0.0008, 0.025)	0.036
** Middle-income**	0.0006 (-0.015, 0.016)	0.936	-0.0031 (-0.015, 0.009)	0.622	-0.0001 (-0.014, 0.013)	0.986
** Upper-lower-income**	-0.007 (-0.352, 0.79)	0.542	-0.0004 (-0.018, 0.17)	0.964	0.011 (-0.008, 0.030)	0.266
** Lower-income**	-0.001 (-0.043, 0.040)	0.943	-0.026 (0.0002, 0.053)	0.048	0.008 (-0.027, 0.042)	0.667
**Restaurants**						
** Upper-income**	0.001 (-0.0009, 0.004)	0.218	-0.002 (-0.005, -0.0001)	0.038	0.003 (-0.0005, 0.006)	0.096
** Middle-upper-income**	0.0006 (-0.002, 0.003)	0.674	-0.002 (-0.005, 0.0003)	0.027	0.003 (-0.0002, 0.006)	0.070
** Middle-income**	-0.0003 (-0.004, 0.004)	0.895	-0.0006 (-0.004, 0.002)	0.721	0.001 (-0.002, 0.005)	0.381
** Upper-lower-income**	-0.001 (-0.005, 0.003)	0.622	-0.001 (-0.005, 0.002)	0.509	0.005 (0.002, 0.009)	0.005
** Lower-income**	0.004 (0.008, 0.017)	0.479	0.005 (-0.003, 0.013)	0.266	0.017 (0.007, 0.027)	0.001
**Supermarkets**						
** Upper-income**	0.037 (-0.022, 0.096)	0.220	-0.034 (-0.088, 0.021)	0.225	-0.074 (-0.149, 0.00007)	0.050
** Middle-upper-income**	0.002 (-0.070, 0.073)	0.962	-0.067 (-0.124, -0.010)	0.021	0.038 (-0.037, 0.114)	0.322
** Middle-income**	-0.016 (-0.109, 0.077)	0.741	-0.030 (-0.102, 0.042)	0.412	0.095 (0.013, 0.177)	0.023
** Upper-lower-income**	-0.053 (-0.170, 0.063)	0.371	0.108 (0.014, 0.203)	0.025	0.041 (-0.061, 0.149)	0.455
** Lower-income**	0.081 (-0.087, 0.250)	0.345	-0.184 (-0.29, -0.076)	0.001	0.059 (-0.084, 0.201)	0.421
**Convenience stores**						
** Upper-income**	-0.006 (-0.023, 0.011)	0.493	0.005 (-0.010, 0.021)	0.495	0.007 (-0.014, 0.029)	0.510
** Middle-upper-income**	0.004 (-0.012, 0.020)	0.634	-0.010 (-0.024, 0.004)	0.158	0.012 (-0.006, 0.030)	0.194
** Middle-income**	-0.007 (-0.028, 0.014)	0.527	0.003 (-0.014, 0.020)	0.737	0.010 (-0.009, 0.028)	0.301
** Upper-lower-income**	0.003 (-0.021, 0.027)	0.824	0.023 (0.003, 0.043)	0.025	-0.001 (-0.023, 0.020)	0.903
** Lower-income**	0.010 (-0.052, 0.072)	0.744	0.069 (0.029, 0.011)	0.001	-0.020 (-0.072, 0.031)	0.443

SEP: socioeconomic position, quintiles: lowest, second lowest, middle, second highest and highest.

^a^ Model A, adjusted by age and sex, and stratified by SEP.

Bold values indicate statistically significant values (p < 0.05)

A low fast-food outlet density was associated with lower-income (β: -0.061, 95% CI: -0.072, -0.050, P < 0.001) and upper-lower-income households (β: -0.026, 95% CI: -0.034, -0.018, P < 0.001). Similarly, a low density of restaurants and supermarkets was associated with low-income neighbourhoods, but a graded association showed that as income increased, a higher availability of restaurants and supermarkets became available to higher-income sectors of the population. Convenience stores were widely available in all socioeconomic strata (Table 4). No statistically significant associations were identified when testing the interaction between socioeconomic position and the density of each of the food outlets tested in this study.

Regarding the association of dietary patterns and food outlet density, stratified by household SEP, when exposed to a high density of fast-food outlets, middle-upper-income populations were associated with the consumption of a carbohydrates-and-drinks type of dietary (β: 0.013, 95% CI 0.0008, 0.025, P = 0.036). Whereas middle-upper-income (β: -0.009, 95% CI -0.019, -0.0003, P = 0.043) and lower-income populations (β: -0.026, 95% CI 0.0002, 0.053, P = 0.036) were associated with an unhealthy dietary pattern (Table 5). Regarding restaurants, lower-income (β: 0.017, 95% CI: 0.007, 0.027, P = 0.001) and upper-lower-income households (β: 0.005, 95% CI: 0.002, 0.009, P = 0.005) were associated with a dietary pattern rich in carbohydrates and drinks whilst middle-upper-income (β: -0.002, 95% CI: -0.005, 0.0003, P = 0.027) and upper-income households (β: -0.002, 95% CI: -0.005, -0.0001, P = 0.038) were inversely associated with unhealthy dietary patterns when exposed to a high density of restaurants. When exposed to a high density of supermarkets, lower-income neighbourhoods (β: -0.184, 95% CI: -0.29, -0.076, P = 0.001) were inversely associated with unhealthy dietary patterns whilst for upper-lower-income households there was an increased association with consuming an unhealthy dietary patterns (β: 0.108, 95% CI: 0.014, 0.203, P = 0.025) and middle-income households were associated with a carbohydrates-and-drinks type of pattern (β: 0.095, 95% CI: 0.013, 0.177, P = 0.023) (Table 5). In geographical areas with a high concentration of convenience stores, lower-income (β: 0.069, 95% CI: 0.029, 0.011, P = 0.001) and upper-lower-income households (β: 0.023, 95% CI: 0.003, 0.043, P = 0.025) were associated with unhealthy dietary patterns (Table 5).

## Discussion

This study assessed 1) the dietary patterns from the Mexican population according to the ENSANUT survey; 2) the association between food outlet density and dietary patterns; 3) the association of the food environment and BMI a) considering diet as a confounder and b) considering the interaction of the food environment and diet, 4) the association and interaction of food outlet density and SEP and 5) the association of dietary patterns and SEP.

### Food outlet density and dietary pattern association

Fruit and vegetable store density in Mexico was inversely associated with an unhealthy dietary pattern in the Mexican population, which may be indicating that having a low availability of fruit and vegetable stores could influence unhealthy dietary patterns. Other studies that assessed fruit and vegetable store availability and dietary intake have observed similar findings [39, 40]. Ollberding et al. [41], identified that living in areas with a greater healthy food outlet access was associated with a higher mean intake of fruits and vegetables [41]. Another study by Menezes et al. [42], observed that the average consumption of fruit and vegetables was higher in neighbourhoods with higher-income and concentration of food stores, and better access to healthy foods. Additionally, fruit and vegetable consumption is low, particularly in low- and middle-income countries (LMIC) where affordability poses an important barrier [43]. Worldwide policies that enhance the availability and affordability of fruits and vegetables may be key to improve dietary intake [43].

Intervention studies that have focused on increasing fruit and vegetable intake have identified that affordability, palatability, and accessibility are some of the key factors that can influence healthy food selection such as fruits and vegetables. However, many interventions focused on increasing fruit and vegetable availability have mostly identified that fruit, but not vegetable consumption, has increased after interventions [44, 45]. Other studies suggest that greater spatial accessibility to food outlets comprising the local food environment may not guarantee fruit and vegetable consumption. This could be explained by a number of factors including high price, low quality and the availability of unhealthy alternatives including ultra-processed foods and beverages which tend to be more affordable, widely accessible and heavily marketed [46]. Therefore, in addition to the availability of fruit and vegetable stores which offer affordable and good quality foods, the restriction of unhealthy and ultra-processed foods to the population are important elements to consider when aiming to improve dietary intake.

Between 2012 and 2014, Mexico exported $3.8 billion a year worth of fruits and nuts; by 2015–17, Mexican fruit and nut exports increased over 55% to $6 billion a year [47] whilst ultra-processed goods were imported to Mexico [48–50]. As the availability of food outlets selling mostly ultra-processed foods has increased, fruit and vegetable stores availability has not [20, 21]. Therefore, increasing accessibility to fruit and vegetable stores that offer a wide variety and high quality of fruits and vegetables along the regulation of unhealthy food offers to the local population could be an important factor to promote healthy food choices.

Our study also identified that restaurants were associated with a dietary pattern rich in carbohydrates and drinks. Complementary food and a high availability of sugar-sweetened beverages (SSBs) and alcoholic drinks, which are all high in simple carbohydrates are common in restaurant environments. Previous studies have identified that restaurant consumers may consume a higher level of carbohydrates [51], sugar-sweetened beverages [52] and alcohol [51] which relates to a higher caloric intake when eating out of home [51].

Previous interventions have focused on including nutritional information on restaurant menus [53]. However, replacing unhealthy appetizers and complementary foods with healthy alternatives (e.g., fruits, vegetables, pulses, nuts) and increasing the ratio of healthy vs unhealthy options may have a more effective impact on improving and facilitating healthier dietary patterns [53].

### BMI, diet, and food environment

When considering dietary patterns as confounders, we identified that a high density of convenience stores was associated with unhealthy dietary patterns and a higher BMI risk. This result aligns with the results identified on our previous study which showed a significant association between a higher density of convenience stores and a higher risk of obesity [20]. Although a higher availability of healthy food options (e.g., fruit and vegetables) and consumption of healthier dietary patterns may be important for the prevention of obesity and NCDs this does not seem to override the abundant availability of food outlets which offer unhealthy food options such as HFSS and ultra-processed foods. This coincides with findings from other studies which suggest that unhealthy food cues have a larger effect than healthier food. Thus, reducing the availability of unhealthy food outlets and removing or restricting less healthy food choices as opposed to only increasing the availability of healthier food options may have a greater impact on dietary intake and the prevention of obesity and diet related NCDs [54–57].

### Socioeconomic position, dietary patterns, and food outlet density

An inverse association was observed between SEP and fruit and vegetable stores and supermarket availability, indicating low-income households were more likely to inhabit areas with a lower access to fruit and vegetable stores and supermarkets. In addition, when exposed to areas with a high availability of fruit and vegetable stores, upper-income households were more likely to consume healthier dietary patterns whilst upper middle-income households were less likely to consume unhealthy dietary patterns.

Fruit and vegetable stores and supermarkets offer a greater availability and variety of healthy food choices (e.g., fruits and vegetables, whole grain alternatives and low-fat options) compared with fast-food outlets, restaurants, and convenience stores. Therefore, having a lower availability of food stores that offer healthy foods such as fruit and vegetable stores and supermarkets in deprived areas may be key barrier to access healthy and affordable foods. Similar findings have been reported in other middle-income countries such as Brazil, where it was observed that socially disadvantaged neighbourhoods had lower access to fruits and vegetables or were of a lower quality [58].

In contrast, convenience stores, which may offer more unhealthy food and beverage options, were widely available for lower-income populations as well as all other household strata. These type of food stores tend to offer a wide range of foods high in saturated fat, salt and sugar which may increase the risk of obesity and NCDs as shown by previous studies [20, 59].

Regarding fast-food outlet exposure and SEP, lower-income households were less exposed to high concentrations of fast-food outlets (β: -0.061 95%CI: -0.072, -0.050; P < 0.001) and were less likely to consume unhealthy dietary patterns when exposed to high concentrations of fast-food outlets (β: -0.026, 95% CI: 0.0002, 0.053; P = 0.04). This could be explained by the low level of development and commercial infrastructure to which lower-income populations may be exposed to [20, 60, 61]. Our previous study assessed the distribution of food outlets according to the level of urbanicity and it was observed that in areas of higher urbanicity, there was a higher availability of fast-food outlets [20]. Similarly, previous studies have shown that fast-food outlets tend to aggregate in the same geographic areas and locate in areas of greater visibility and connectivity, such as city centres, commercial areas and highstreets which may have a higher street intersection density, a higher availability of public transport and infrastructure that facilitates walkability and safe mobility (e.g., sidewalks, bike lanes, parks) [62]. This strategy helps fast food outlets dominate the market and maximise profits [62] which explains why fast-food outlets in lower-income neighbourhoods were less available for low-income households in this study. Previous studies have also observed that a high availability of fast‐food outlets increases the risk of obesity by discouraging healthy dietary behaviours along an high exposure to unhealthy food outlets which enable unhealthy food options [63].

Similarly, for restaurants, these were less likely to be found in lower-income areas compared with upper-income areas. However, upper-income populations were less likely to acquire unhealthy dietary patterns when highly exposed to unhealthy food outlets whereas lower-income populations who were exposed to fast-food outlets were more likely to consume a diet rich in carbohydrates. This could be explained by higher-income populations having more purchase power, options, and mobility (e.g., car ownership) to acquire food from a diversity of food outlets and may typically be able to travel further to access a greater diversity of food shops and healthier food choices [64, 65]. Supporting this finding, as indicated in the 2019 Pan American Health Organization report [19], ultra-processed foods purchases increased as available money increased. In Mexico, gross domestic product (GDP) per capita increased by 23% between 2009 and 2014.

Supermarkets were less available for lower-income populations; however, when lower-income populations did have access to supermarkets, a lower likelihood of an unhealthy diet was observed. This could be explained by an increased access to staple foods at more affordable prices and an increased availability of fruits and vegetables. Previous studies have observed that lower-income areas are less likely to have access to supermarkets and grocery stores that carry healthy foods compared with predominantly middle- and higher-income areas [16, 66, 67]. However, when exposed to a high density of supermarkets, upper-lower-income populations were more likely to have an unhealthy dietary pattern. This may be explained by a higher purchase power and an increased exposure to purchase snacks and additional discretionary foods [68]. Previous studies have found a correlation between an increase in purchase power unhealthy food purchases in Latin American countries [68].

Middle- and low-income populations may be more vulnerable to unhealthy foods and beverages commonly displayed at cash points which previous research has observed to incentive unhealthy food choices. Point of purchase policy regulations may target smaller portion sizes for unhealthy foods and beverages; unhealthy combo meal-like promotions; and food choice architecture (e.g. placement and marketing) restrictions on HFSS and ultra-processed foods and increased saliency for healthier alternatives [69]. Adoption of these type of policies could contribute significantly to the prevention of obesity and diet-related NCDs [69].

Our findings indicate that the food environment might explain some of the socioeconomic disparities related with dietary intake. Supporting these finding, a study by Pérez-Ferrer et al. indicated that household wealth can be an effect modifier in the association between education and obesity, mainly in women [70]. The study also indicated that as countries like Mexico develop economically, there tends to be a cross-over to higher rates of obesity among socially disadvantaged groups [70]. As our study indicates, a lack of healthy food store availability for the most deprived may increase the susceptibility to unhealthy diets which may increase the risk of obesity and NCDs.

In Mexico, the nutrition transition and an increase in obesity coincided with the NAFTA agreement [50, 71], which may be related to growth in unhealthy food retail outlets [50]. Our findings indicate that environmental and sociodemographic conditions within neighbourhoods may affect dietary behaviours [58]. Additionally, household income can influence access to food products and food stores, thereby making it difficult for low-income families to prioritize the purchase healthy foods, especially when these are more expensive or not as appetising [58].

### Strengths and limitations

The findings presented here should be interpreted with caution. Among the limitations of our study is the use of cross-sectional data, which does not permit examination of how changes in fruit and vegetable store density influenced consumption. Retail food environments are dynamic, and a longitudinal design could help understand the effect of the retail food environment on dietary intake. Similarly, a natural experimental evaluation could have minimized potential bias and provide critical information about the impacts of food retail interventions on dietary intake [72] or obesity [73]. There was a two-year difference between the health and geographic data that were used in this study. However, data-verification two years later found the prevalence, position and type of food store was still accurate, suggesting little change over time. Additionally, due to data confidentiality, dietary intake data were recorded at the centroid of the residents’ CTA as precise individual level area data were not available. CTA was used to calculate food outlet density as a proxy for individual’s food environment. CTA has been considered a gold standard for measuring food environment and has been used by various studies as the unit of analysis to study food environments [74, 75]. However, individuals often cross the boundaries of their residential area to access food which may underestimate food availability [74]. Additionally, residents in impoverished areas may have limited capital resources, such as car ownership, making it feasible to assume that there may be greater reliance on proximal food sources [76] or low-income populations may travel long distances for work during the day and thus shift across food environments. Additionally, even if healthier food options are available, food affordability may be a barrier if working or commuting via high-income areas. Missing data could have impacted the association of the food environment, diet, obesity, and SEP. ENSANUT carried out a dietary assessment to 11% of the population [26]; therefore, the study’s sample size was restricted to participants who had a measure of dietary intake and the retail food environment. Individuals who did not have a measure of diet, lived in a rural area, or did not have a measure of the retail food environment were excluded from this study.

Although informal food vendors were identified in the food outlet data verification stage, it is possible that some mobile food units, which represent an important influence on dietary intake in Mexico [77], may not have been included in the food outlet database used in this study, which could have been due to lack of registration compliance to sell food or because of the provision of a home address rather than the place of sale. A lack of data on the affordability and quality of fruit and vegetables sold and the availability of unhealthy alternatives is a limitation of this study. To account for this limitation, household SEP and dietary intake in the form of dietary patterns were included in the statistical models. Lastly, due to the self-reporting nature of FFQ questionnaires to assess dietary intake, bias may occur due to underreporting of food consumption or low accuracy (recall bias). In addition, because an FFQ is composed of a specific list of food items, a single FFQ may not reflect the consumption patterns of a given population [78].

In terms of strengths, this study included a comprehensive dietary assessment which permitted examination of the diet through dietary patterns. Dietary patterns may be a better predictor of dietary intake than individual nutrient analysis because they consider the overall diet consumption and account for the interaction of nutrients within foods and their potential effect on health [79]. Additionally, we used principal component and factor analysis to determine dietary patterns, which is the most common approach to determine dietary patterns and therefore allows comparison with other studies. Instead of classifying individuals into a single pattern, individuals receive a score of each pattern instead of being subjectively classified into a single cluster or group [79]. Other strengths include the use of measured data; the geographical location verification of food outlets; accounting for selection bias; and the use of a statistical method that was able to detect discrepancies between different geographical levels and account for clustering. To our knowledge, this is the only study to has assessed the relationship between food retailer availability, dietary patterns, BMI and its interaction with diet, and socioeconomic position at a national level in a middle-income country setting. This study advances the existing literature of the retail food environment and its relationship with food choice and the application of geographical information systems.

## Conclusions

A lower density of fruit and vegetable stores was associated with unhealthier dietary patterns whilst a high exposure to convenience stores was linked with unhealthy dietary patterns and a higher risk of BMI particularly in low-income populations. Actions to regulate the food environment by decreasing the availability of unhealthy foods and increasing the availability of healthy food and beverage options, particularly in low-income neighbourhoods, may help improve dietary intake in the Mexican population and contribute to the prevention of obesity and diet related NCDs in the country.

## Supporting information

S1 Checklist Inclusivity in global research. Additional information regarding the ethical, cultural, and scientific considerations specific to inclusivity in global research.
(DOCX)

S1 Fig Scree plot of eigen values for selection of dietary patterns. (PDF)

S1 Table Dietary patterns, factor loadings, proportion of variance explained and Cronbach’s alpha. A value of ≥0.28 indicates an association between the food group and the factor. Bold factors indicate an association.
(DOCX)

S2 Table Association of the food environment with BMI and the interaction of dietary patterns and the food environment. (DOCX)

S3 Table Association of the food environment and BMI whilst considering dietary patterns as a confounder. (DOCX)

S4 Table Association of the food environment and obesity accounting for dietary patterns and stratifying by sex. (DOCX)

## References

[1] Butland B, Jebb S, Kopelman P, McPherson K, Thomas S, Mardell J, et al. FORESIGHT—Tackling Obesities: future Choices—Project Report. UK: 2007. 10.1111/j.1467-789X.2007.00344.x 17316292

[2] EUFIC. The determinants of food choice Belgium: European Food Information Council; 2005 [cited 2015 22/08/2015]. Available from: http://www.eufic.org/article/en/expid/review-food-choice/.

[3] Branca F, Lartey A, Oenema S, Aguayo V, Stordalen GA, Richardson R, et al. Transforming the food system to fight non-communicable diseases. BMJ. 2019;364:l296. doi: 10.1136/bmj.l296 30692128 PMC6349221

[4] Vepsäläinen H, Mikkilä V, Erkkola M, Broyles ST, Chaput JP, Hu G, et al. Association between home and school food environments and dietary patterns among 9-11-year-old children in 12 countries. Int J Obes Suppl. 2015;5(Suppl 2):S66–73. Epub 2016/05/07. doi: 10.1038/ijosup.2015.22 ; PubMed Central PMCID: PMC4850623. 27152188 PMC4850623

[5] Stafford M, Cummins S, Ellaway A, Sacker A, Wiggins RD, Macintyre S. Pathways to obesity: Identifying local, modifiable determinants of physical activity and diet. Soc Sci Med. 2007;65(9):1882–97. doi: 10.1016/j.socscimed.2007.05.042 17640787

[6] Glanz K, Sallis JF, Saelens BE, Frank LD. Healthy Nutrition Environments: Concepts and Measures. American Journal of Health Promotion. 2005;19(5):330–3. doi: 10.4278/0890-1171-19.5.330 . 15895534

[7] Swinburn B, Kraak V, Rutter H, Vandevijvere S, Lobstein T, Sacks G, et al. Strengthening of accountability systems to create healthy food environments and reduce global obesity. Lancet. 2015;385(9986):2534–45. Epub 2015/02/24. doi: 10.1016/S0140-6736(14)61747-5 . 25703108

[8] Office of the United States Trade Representative. North American Free Trade Agreement (NAFTA) US2017 [cited 2020 2 March]. Available from: https://ustr.gov/trade-agreements/free-trade-agreements/north-american-free-trade-agreement-nafta.

[9] Friel S, Hattersley L, Snowdon W, Thow AM, Lobstein T, Sanders D, et al. Monitoring the impacts of trade agreements on food environments. Obes Rev. 2013;14 Suppl 1:120–34. doi: 10.1111/obr.12081 . 24074216

[10] Imamura F, Micha R, Khatibzadeh S, Fahimi S, Shi P, Powles J, et al. Dietary quality among men and women in 187 countries in 1990 and 2010: a systematic assessment. The Lancet Global Health. 2015;3(3):e132–e42. doi: 10.1016/S2214-109X(14)70381-X 25701991 PMC4342410

[11] Hawkes C. Uneven dietary development: linking the policies and processes of globalization with the nutrition transition, obesity and diet-related chronic diseases. Global Health. 2006;2:4. Epub 2006/03/30. doi: 10.1186/1744-8603-2-4 ; PubMed Central PMCID: PMC1440852. 16569239 PMC1440852

[12] INSP. Encuesta Nacional de Salud y Nutrición 2018 [2018 Health and Nutrition National Survey]. Mexico: Instituto Nacional de Salúd Pública [National Health Insitute], 2018.

[13] Colchero MA, Molina M, Guerrero-López CM. After Mexico Implemented a Tax, Purchases of Sugar-Sweetened Beverages Decreased and Water Increased: Difference by Place of Residence, Household Composition, and Income Level. The Journal of nutrition. 2017;147(8):1552–7. Epub 2017/06/14. doi: 10.3945/jn.117.251892 . 28615377 PMC5525113

[14] PAHO. Alimentos y bebidas ultraprocesados en América Latina: ventas, fuentes, perfiles de nutrientes e implicaciones normativas [Ultraprocessed foods and beverages in Latin America: sales, sources, nutritional profiles and normative implications]. Washington, DC: PAHO, 2019.

[15] INSP. Aspectos económicos relacionados con un impuesto al refresco en México [Economic aspects related to the soda tax in Mexico]. Mexico: 2013.

[16] Institute of Medicine (US) and National Research Council (US) Committee on Childhood Obesity. Local Government Actions to Prevent Childhood Obesity Washington D.C.: National Academies Press; 2009 [cited 2022 February]. Available from: https://www.ncbi.nlm.nih.gov/books/NBK219682/.

[17] Institute OD. The rising cost of a healthy diet—Changing relative prices of foods in high-income and emerging economies. London, UK: Overseas Development Institute, 2015.

[18] Marrón-Ponce JA, Tolentino-Mayo L, Hernández-F M, Batis C. Trends in Ultra-Processed Food Purchases from 1984 to 2016 in Mexican Households. Nutrients. 2018;11(1):45. doi: 10.3390/nu11010045 . 30587779 PMC6356651

[19] Pan American Health Organization. Ultra-processed food and drink products in Latin America: Sales, sources, nutrient profiles and policy implications. Washington, D. C.: PAHO, 2019.

[20] Pineda E, Brunner EJ, Llewellyn CH, Mindell JS. The retail food environment and its association with body mass index in Mexico. Int J Obes (Lond). 2021;45(6):1215–28. Epub 2021/02/19. doi: 10.1038/s41366-021-00760-2 ; PubMed Central PMCID: PMC8159738. 33597735 PMC8159738

[21] Pérez-Ferrer C, Auchincloss AH, Barrientos-Gutierrez T, Colchero MA, de Oliveira Cardoso L, Carvalho de Menezes M, et al. Longitudinal changes in the retail food environment in Mexico and their association with diabetes. Health Place. 2020;66:102461. Epub 2020/10/12. doi: 10.1016/j.healthplace.2020.102461 ; PubMed Central PMCID: PMC7705211. 33039800 PMC7705211

[22] Shaw SC, Ntani G, Baird J, Vogel CA. A systematic review of the influences of food store product placement on dietary-related outcomes. Nutr Rev. 2020;78(12):1030–45. doi: 10.1093/nutrit/nuaa024 32483615 PMC7666915

[23] Krukowski RA, West DS, Harvey-Berino J, Elaine Prewitt T. Neighborhood impact on healthy food availability and pricing in food stores. J Community Health. 2010;35(3):315–20. doi: 10.1007/s10900-010-9224-y . 20127506 PMC3071013

[24] Drewnowski A, Moudon AV, Jiao J, Aggarwal A, Charreire H, Chaix B. Food environment and socioeconomic status influence obesity rates in Seattle and in Paris. Int J Obes (Lond). 2014;38(2):306–14. doi: 10.1038/ijo.2013.97 23736365 PMC3955164

[25] INSP. National Health and Nutrition Survey [Encuesta Nacional de Salud y Nutrición]. Mexico: INSP, 2012.

[26] INSP. ENSANUT 2012 Encuesta Nacional de Salud y Nutrición 2012. Mexico: INSP, 2012.

[27] STATA. Your data tell a story 2022 [cited 2022 June]. Available from: https://www.stata.com/.

[28] World Health O. Recomendaciones mundiales sobre actividad física para la salud. Ginebra: Organización Mundial de la Salud; 2010.

[29] INEGI. Encuesta Nacional de Ingresos y Gastos de los Hogares (ENIGH) [National Income and Expenditure Survey 2010] Mexico: Instituto Nacional de Estadística y Geografía [National Institute of Statistics and Geography]; 2010 [cited 2022]. Available from: https://www.inegi.org.mx/rnm/index.php/catalog/36.

[30] Denova-Gutiérrez E., Ramírez-Silva I., Rodríguez-Ramírez S., Jiménez-Aguilar A., Shamah-Levy T., R-D J.A. Validity of a food frequency questionnaire to assess food intake in Mexican adolescent and adult population Mexico: INSP; 2016 [cited 2022 October]. Available from: https://www.saludpublica.mx/index.php/spm/article/view/7862. 10.21149/spm.v58i6.7862 28225938

[31] INEGI. Censos económicos 2014 Mexico: Instituto Nacional de Estadística y Geografía; 2017 [cited 2017 25/Septebmer]. Available from: http://www.inegi.org.mx/.

[32] Caspi CE, Friebur R. Modified ground-truthing: an accurate and cost-effective food environment validation method for town and rural areas. The international journal of behavioral nutrition and physical activity. 2016;13:37–. doi: 10.1186/s12966-016-0360-3 . 26988710 PMC4794836

[33] Pérez-Tepayo S, Rodríguez-Ramírez S, Unar-Munguía M, Shamah-Levy T. Trends in the dietary patterns of Mexican adults by sociodemographic characteristics. Nutr J. 2020;19(1):51. doi: 10.1186/s12937-020-00568-2 32460758 PMC7254758

[34] Valerino-Perea S, Lara-Castor L, Armstrong MEG, Papadaki A. Definition of the Traditional Mexican Diet and Its Role in Health: A Systematic Review. Nutrients. 2019;11(11). Epub 2019/11/21. doi: 10.3390/nu11112803 ; PubMed Central PMCID: PMC6893605. 31744179 PMC6893605

[35] Cunha DB, Almeida RM, Pereira RA. A comparison of three statistical methods applied in the identification of eating patterns. Cadernos de saude publica. 2010;26(11):2138–48. Epub 2010/12/25. doi: 10.1590/s0102-311x2010001100015 . 21180987

[36] Consonni G, Leucari V. Model Determination for Directed Acyclic Graphs. Journal of the Royal Statistical Society Series D (The Statistician). 2001;50(3):243–56.

[37] CONAPO S, SEGO B. Catálogo Sistema Urbano Nacional 2012 Mexico: CONAPO; 2012 [cited 2022 August]. Available from: http://www.conapo.gob.mx/work/models/CONAPO/Resource/1539/1/images/PartesIaV.pdf.

[38] Hair JF. Multivariate data analysis. Upper Saddle River, N.J.: Prentice Hall; 1998.

[39] Franco M, Diez-Roux AV, Nettleton JA, Lazo M, Brancati F, Caballero B, et al. Availability of healthy foods and dietary patterns: the Multi-Ethnic Study of Atherosclerosis. The American Journal of Clinical Nutrition. 2009;89(3):897–904. doi: 10.3945/ajcn.2008.26434 19144728 PMC2667662

[40] Pitts SBJ, Gustafson A, Wu Q, Mayo ML, Ward RK, McGuirt JT, et al. Farmers’ market use is associated with fruit and vegetable consumption in diverse southern rural communities. Nutr J. 2014;13(1):1. doi: 10.1186/1475-2891-13-1 24405527 PMC3896848

[41] Ollberding NJ, Nigg CR, Geller KS, Horwath CC, Motl RW, Dishman RK. Food outlet accessibility and fruit and vegetable consumption. Am J Health Promot. 2012;26(6):366–70. Epub 2012/07/04. doi: 10.4278/ajhp.101215-ARB-401 . 22747319

[42] Menezes MC, Costa BVL, Oliveira CDL, Lopes ACS. Local food environment and fruit and vegetable consumption: An ecological study. Preventive Medicine Reports. 2017;5:13–20. doi: 10.1016/j.pmedr.2016.10.015 27872803 PMC5114690

[43] Miller V, Yusuf S, Chow CK, Dehghan M, Corsi DJ, Lock K, et al. Availability, affordability, and consumption of fruits and vegetables in 18 countries across income levels: findings from the Prospective Urban Rural Epidemiology (PURE) study. The Lancet Global Health. 2016;4(10):e695–e703. doi: 10.1016/S2214-109X(16)30186-3 27567348

[44] Appleton KM, Hemingway A, Saulais L, Dinnella C, Monteleone E, Depezay L, et al. Increasing vegetable intakes: rationale and systematic review of published interventions. Eur J Nutr. 2016;55(3):869–96. Epub 2016/01/13. doi: 10.1007/s00394-015-1130-8 ; PubMed Central PMCID: PMC4819941. 26754302 PMC4819941

[45] Perry CL, Bishop DB, Taylor G, Murray DM, Mays RW, Dudovitz BS, et al. Changing fruit and vegetable consumption among children: the 5-a-Day Power Plus program in St. Paul, Minnesota. Am J Public Health. 1998;88(4):603–9. doi: 10.2105/ajph.88.4.603 . 9551002 PMC1508423

[46] Popkin BM, Adair LS, Ng SW. Global nutrition transition and the pandemic of obesity in developing countries. Nutr Rev. 2012;70(1):3–21. doi: 10.1111/j.1753-4887.2011.00456.x . 22221213 PMC3257829

[47] Dialogue M. Rising Fruit and Vegetables Imports from Mexico: UC Davis; 2018 [cited 2020 September]. Available from: https://migration.ucdavis.edu/.

[48] USDA. Fruit and vegetables USA: USDA; 2020. Available from: https://www.ers.usda.gov/data-products/food-availability-per-capita-data-system/.

[49] Mendoza-Cano O, Sánchez-Piña RA, González-Ibarra Á J, Murillo-Zamora E, Nava-Garibaldi CM. Health Impacts from Corn Production Pre-and Post-NAFTA Trade Agreement (1986–2013). Int J Environ Res Public Health. 2016;13(7). Epub 2016/07/16. doi: 10.3390/ijerph13070709 ; PubMed Central PMCID: PMC4962250. 27420088 PMC4962250

[50] Clark SE, Hawkes C, Murphy SM, Hansen-Kuhn KA, Wallinga D. Exporting obesity: US farm and trade policy and the transformation of the Mexican consumer food environment. International journal of occupational and environmental health. 2012;18(1):53–65. Epub 2012/05/04. doi: 10.1179/1077352512Z.0000000007 . 22550697

[51] Orfanos P, Naska A, Rodrigues S, Lopes C, Freisling H, Rohrmann S, et al. Eating at restaurants, at work or at home. Is there a difference? A study among adults of 11 European countries in the context of the HECTOR* project. Eur J Clin Nutr. 2017;71(3):407–19. doi: 10.1038/ejcn.2016.219 27966568

[52] Ayala GX, Rogers M, Arredondo EM, Campbell NR, Baquero B, Duerksen SC, et al. Away-from-home food intake and risk for obesity: examining the influence of context. Obesity (Silver Spring, Md). 2008;16(5):1002–8. Epub 2008/02/28. doi: 10.1038/oby.2008.34 . 18309297 PMC3758884

[53] Mandracchia F, Tarro L, Llauradó E, Valls RM, Solà R. Interventions to Promote Healthy Meals in Full-Service Restaurants and Canteens: A Systematic Review and Meta-Analysis. Nutrients. 2021;13(4):1350. doi: 10.3390/nu13041350 . 33919552 PMC8073122

[54] Pechey R, Marteau TM. Availability of healthier vs. less healthy food and food choice: an online experiment. BMC Public Health. 2018;18(1):1296. doi: 10.1186/s12889-018-6112-3 30486801 PMC6264049

[55] Rose D, Hutchinson PL, Bodor JN, Swalm CM, Farley TA, Cohen DA, et al. Neighborhood Food Environments and Body Mass Index: The Importance of In-Store Contents. Am J Prev Med. 2009;37(3):214–9. doi: 10.1016/j.amepre.2009.04.024 19666158 PMC2945712

[56] Nakamura R, Suhrcke M, Jebb SA, Pechey R, Almiron-Roig E, Marteau TM. Price promotions on healthier compared with less healthy foods: a hierarchical regression analysis of the impact on sales and social patterning of responses to promotions in Great Britain. The American Journal of Clinical Nutrition. 2015;101(4):808–16. doi: 10.3945/ajcn.114.094227 25833978 PMC4381774

[57] Talukdar D, Lindsey C. To Buy or Not to Buy: Consumers’ Demand Response Patterns for Healthy versus Unhealthy Food. Journal of Marketing. 2013;77(2):124–38. doi: 10.1509/jm.11.0222

[58] Pessoa MC, Mendes LL, Gomes CS, Martins PA, Velasquez-Melendez G. Food environment and fruit and vegetable intake in a urban population: a multilevel analysis. BMC Public Health. 2015;15:1012. Epub 2015/10/07. doi: 10.1186/s12889-015-2277-1 ; PubMed Central PMCID: PMC4595198. 26437719 PMC4595198

[59] Xin J, Zhao L, Wu T, Zhang L, Li Y, Xue H, et al. Association between access to convenience stores and childhood obesity: A systematic review. Obes Rev. 2021;22(S1):e12908. doi: 10.1111/obr.12908 31274248 PMC7988541

[60] Ziccardi A. Poverty and urban inequality: the case of Mexico City metropolitan region*. International Social Science Journal. 2014;65(217–218):205–19. 10.1111/issj.12070.

[61] Capron G, Gonzalez Arellano S, Wigle J, Diez AL, Monterrubio A, Castro J, et al. The urban food system of Mexico City, Mexico University of Cape Town, South Africa: Hungry cities partnership; 2017 [cited 2022 February]. Available from: https://hungrycities.net/wp-content/uploads/2017/12/MexicoReport.pdf.

[62] Thomadsen R. Product Positioning and Competition: The Role of Location in the Fast Food Industry. Marketing Science. 2007;26(6):792–804.

[63] Jia P, Luo M, Li Y, Zheng JS, Xiao Q, Luo J. Fast‐food restaurant, unhealthy eating, and childhood obesity: A systematic review and meta‐analysis. Obes Rev. 2019;22. doi: 10.1111/obr.12944 31507064 PMC7988557

[64] Pechey R, Monsivais P. Socioeconomic inequalities in the healthiness of food choices: Exploring the contributions of food expenditures. Prev Med. 2016;88:203–9. Epub 2016/04/16. doi: 10.1016/j.ypmed.2016.04.012 . 27095324 PMC4910945

[65] Gustat J, O’Malley K, Luckett BG, Johnson CC. Fresh produce consumption and the association between frequency of food shopping, car access, and distance to supermarkets. Preventive Medicine Reports. 2015;2:47–52. doi: 10.1016/j.pmedr.2014.12.009 26844049 PMC4721283

[66] Baker EA, Schootman M, Barnidge E, Kelly C. The role of race and poverty in access to foods that enable individuals to adhere to dietary guidelines. Prev Chronic Dis. 2006;3(3):A76–A. Epub 2006/06/15. . 16776877 PMC1636719

[67] Franco M, Diez Roux AV, Glass TA, Caballero B, Brancati FL. Neighborhood characteristics and availability of healthy foods in Baltimore. Am J Prev Med. 2008;35(6):561–7. Epub 2008/10/08. doi: 10.1016/j.amepre.2008.07.003 . 18842389 PMC4348113

[68] Popkin BM, Reardon T. Obesity and the food system transformation in Latin America. Obes Rev. 2018;19(8):1028–64. doi: 10.1111/obr.12694 29691969 PMC6103889

[69] Cohen DA, Lesser LI. Obesity prevention at the point of purchase. Obesity reviews: an official journal of the International Association for the Study of Obesity. 2016;17(5):389–96. Epub 2016/02/22. doi: 10.1111/obr.12387 . 26910361 PMC5406228

[70] Perez-Ferrer C, McMunn A, Zaninotto P, Brunner EJ. The nutrition transition in Mexico 1988–2016: the role of wealth in the social patterning of obesity by education. Public Health Nutr. 2018;21(13):2394–401. Epub 2018/05/11. doi: 10.1017/S1368980018001167 . 29745353 PMC10260958

[71] Siegel AD. NAFTA Largely Responsible for the Obesity Epidemic in Mexico. Washington University Journal of Law and Policy. 2016;50:195–226.

[72] Sadler RC, Gilliland JA, Arku G. A food retail-based intervention on food security and consumption. International journal of environmental research and public health. 2013;10(8):3325–46. doi: 10.3390/ijerph10083325 . 23921626 PMC3774441

[73] Taillie LS, Grummon AH, Fleischhacker S, Grigsby-Toussaint DS, Leone L, Caspi CE. Best practices for using natural experiments to evaluate retail food and beverage policies and interventions. Nutr Rev. 2017;75(12):971–89. doi: 10.1093/nutrit/nux051 . 29190370 PMC6280926

[74] Charreire H, Casey R, Salze P, Simon C, Chaix B, Banos A, et al. Measuring the food environment using geographical information systems: a methodological review. Public Health Nutr. 2010;13(11):1773–85. Epub 2010/04/23. doi: 10.1017/S1368980010000753 . 20409354

[75] Ma X, Battersby SE, Bell BA, Hibbert JD, Barnes TL, Liese AD. Variation in low food access areas due to data source inaccuracies. Appl Geogr. 2013;45:10.1016/j.apgeog.2013.08.014. doi: 10.1016/j.apgeog.2013.08.014 . 24367136 PMC3869099

[76] Lee RE, Heinrich KM, Medina AV, Regan GR, Reese-Smith JY, Jokura Y, et al. A picture of the healthful food environment in two diverse urban cities. Environ Health Insights. 2010;4:49–60. doi: 10.4137/ehi.s3594 . 20706621 PMC2918357

[77] Long-Solís J. A Survey of Street Foods in Mexico City. Food and Foodways. 2007;15(3–4):213–36. doi: 10.1080/07409710701620136

[78] Shim JS, Oh K, Kim HC. Dietary assessment methods in epidemiologic studies. Epidemiol Health. 2014;36:e2014009. Epub 2014/08/01. doi: 10.4178/epih/e2014009 ; PubMed Central PMCID: PMC4154347. 25078382 PMC4154347

[79] Schulze MB, Martínez-González MA, Fung TT, Lichtenstein AH, Forouhi NG. Food based dietary patterns and chronic disease prevention. BMJ. 2018;361:k2396–k. doi: 10.1136/bmj.k2396 . 29898951 PMC5996879

