# Supplementary figures and images for: Association of the retail food environment, BMI, dietary patterns, and socioeconomic position in urban areas of Mexico
Source: PLOS Glob Public Health. 2023 Feb 23;3(2):e0001069. doi: 10.1371/journal.pgph.0001069 (PMC10022358; doi:10.1371/journal.pgph.0001069)

Supplementary Figure S1. Scree plot of eigenvalues

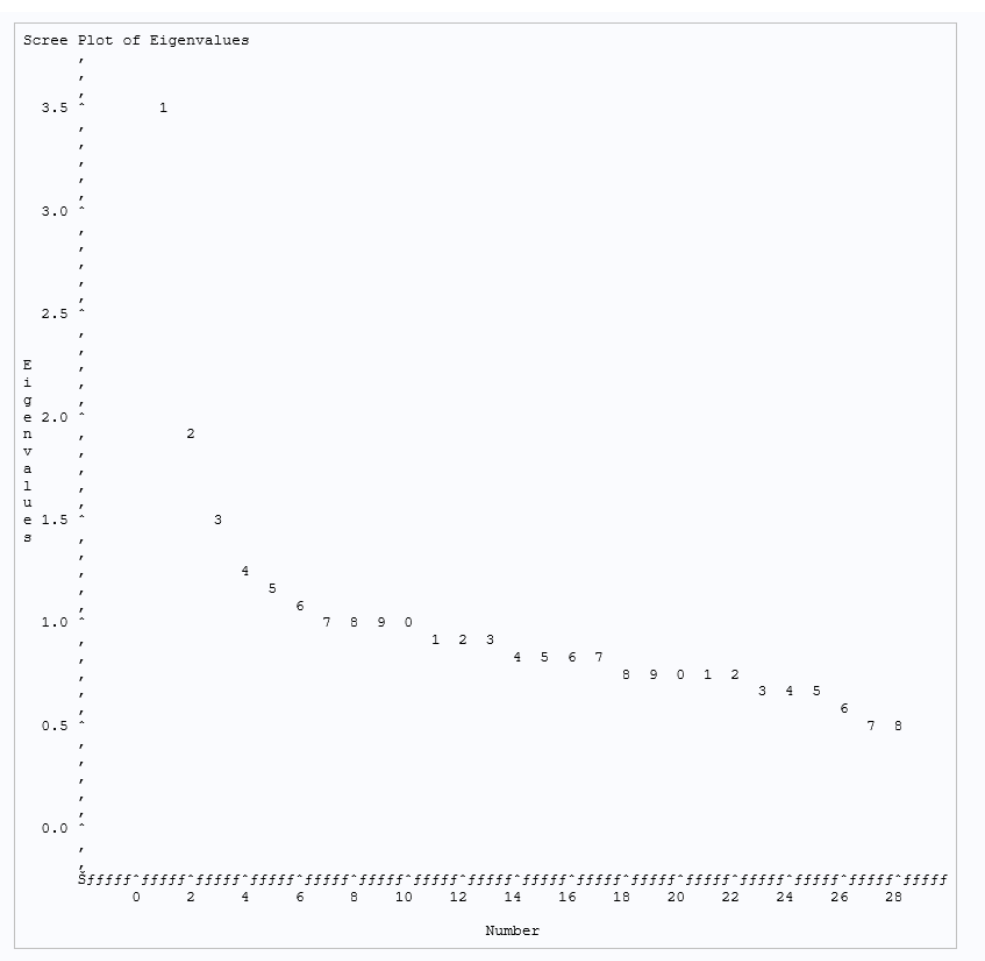

Supplement: S1 Fig — (PDF) [file pgph.0001069.s002.pdf]
