# Supplementary material for: Association of the retail food environment, BMI, dietary patterns, and socioeconomic position in urban areas of Mexico
Source: PLOS Glob Public Health. 2023 Feb 23;3(2):e0001069. doi: 10.1371/journal.pgph.0001069 (PMC10022358; doi:10.1371/journal.pgph.0001069)
Supplement: S1 Table — A value of ≥0.28 indicates an association between the food group and the factor. Bold factors indicate an association. (DOCX) [file pgph.0001069.s003.docx]

**S1 Table. Dietary patterns, factor loadings, proportion of variance explained and Cronbach’s alpha**

|  | Food groups | Factor 1  Healthy pattern | Factor 2  Unhealthy pattern | Factor 3  Carbohydrate & drinks pattern |
| --- | --- | --- | --- | --- |
| 1 | Vegetables | **0.72** | 0.06 | 0.06 |
| 2 | Lemon and chilli | **0.53** | 0.22 | -0.25 |
| 3 | Meat (e.g. pork, beef, chicken) | **0.49** | 0.19 | 0.05 |
| 4 | Cooked meals (e.g. rice, stews with vegetables) | **0.48** | 0.05 | 0.09 |
| 5 | Fruit | **0.45** | -0.04 | 0.42 |
| 6 | Bread, crackers, and potatoes | **0.43** | 0.14 | 0.02 |
| 7 | Soups | **0.43** | 0.04 | 0.08 |
| 8 | Fermented dairy (cheese and yogurt) | **0.34** | 0.27 | 0.24 |
| 9 | Fats | 0.31 | **0.33** | -0.11 |
| 10 | Juice and natural drinks | **0.30** | -0.07 | **0.36** |
| 11 | Fried beans and pulses | **0.28** | 0.24 | -0.12 |
| 12 | Fish and seafood | **0.28** | 0.02 | 0.15 |
| 13 | Beans and pulses in water | 0.25 | -0.24 | -0.18 |
| 14 | Coffee (with and without sugar) | 0.23 | -0.05 | -0.21 |
| 15 | High meat and fatty meals | 0.21 | **0.29** | -0.07 |
| 16 | Tea (with and without sugar) | 0.20 | -0.12 | 0.03 |
| 17 | Sugar and desserts | 0.19 | **0.51** | 0.34 |
| 18 | Sausages | 0.19 | **0.43** | 0.04 |
| 19 | Water | 0.17 | -0.04 | 0.02 |
| 20 | Whole wheat products | 0.13 | -0.02 | **0.33** |
| 21 | Milk | 0.09 | 0.11 | **0.63** |
| 22 | Alcohol | 0.08 | 0.15 | -0.25 |
| 23 | Dressings | 0.02 | **0.59** | 0.018 |
| 24 | Soda | 0.01 | **0.59** | -0.172 |
| 25 | Fast-food (burger, pizza, hot-dog) | -0.01 | **0.55** | 0.20 |
| 26 | Refined cereal | -0.02 | 0.21 | **0.53** |
| 27 | Ready-to-eat soups | -0.04 | **0.39** | -0.01 |
| 28 | Potato chips and candy | -0.08 | **0.64** | 0.09 |

The factor loadings indicate the correlation between the food group and the factor. Bold factors indicate factor loadings ≥0.28. Proportion of variance explained and Cronbach’s alpha: 26.3% for factor 1; 26.1% for factor 2; and 16.4% for factor 3 of the total variance of the data.
