# Supplementary material for: Association of the retail food environment, BMI, dietary patterns, and socioeconomic position in urban areas of Mexico
Source: PLOS Glob Public Health. 2023 Feb 23;3(2):e0001069. doi: 10.1371/journal.pgph.0001069 (PMC10022358; doi:10.1371/journal.pgph.0001069)
Supplement: S2 Table — (DOCX) [file pgph.0001069.s004.docx]

**S2 Table. Association of the food environment with BMI and the interaction of dietary patterns and the food environment**

| **Model A** |  |  |  |  |
| --- | --- | --- | --- | --- |
| **Factor 1** |  |  |  |  |
| **BMI** | **β** | **95% LCI** | **95% UCI** | **P-value** |
| Factor 1 | -0.14 | -0.60 | 0.32 | 0.54 |
| Convenience stores | 0.00 | -0.01 | 0.01 | 0.92 |
| Interaction Factor 1 & Convenience stores | 0.00 | -0.01 | 0.01 | 0.64 |
|  |  |  |  |  |
| BMI | β | 95% LCI | 95% UCI | P-value |
| Factor 1 | -0.14 | -0.50 | 0.21 | 0.42 |
| Fast-food outlets | 0.03 | -0.01 | 0.07 | 0.17 |
| Interaction Factor 1 & Fast-food outlets | -0.02 | -0.06 | 0.03 | 0.42 |
|  |  |  |  |  |
| BMI | β | 95% LCI | 95% UCI | P-value |
| Factor 1 | -0.21 | -0.57 | 0.16 | 0.27 |
| Restaurants | 0.00 | -0.01 | 0.01 | 0.73 |
| Interaction Factor 1 & Restaurants | 0.00 | -0.01 | 0.01 | 0.83 |
|  |  |  |  |  |
| BMI | β | 95% LCI | 95% UCI | P-value |
| Factor 1 | -0.18 | -0.48 | 0.12 | 0.24 |
| Supermarkets | -0.12 | -0.36 | 0.11 | 0.30 |
| Interaction Factor 1 & Supermarkets | -0.10 | -0.31 | 0.11 | 0.34 |
|  |  |  |  |  |
| BMI | β | 95% LCI | 95% UCI | P-value |
| Factor 1 | -0.16 | -0.46 | 0.13 | 0.28 |
| Fruit and vegetable stores | 0.01 | -0.01 | 0.03 | 0.22 |
| Interaction Factor 1 & Fruit and vegetable stores | -0.01 | -0.03 | 0.00 | 0.12 |
|  |  |  |  |  |
| **Factor 2** |  |  |  |  |
| BMI | β | 95% LCI | 95% UCI | P-value |
| Factor 2 | 0.11 | -0.47 | 0.69 | 0.72 |
| Convenience stores | 0.00 | -0.01 | 0.01 | 0.54 |
| Interaction Factor 2 & Convenience stores | -0.01 | -0.02 | 0.00 | 0.10 |
|  |  |  |  |  |
| BMI | β | 95% LCI | 95% UCI | P-value |
| Factor 2 | 0.00 | -0.46 | 0.45 | 0.99 |
| Fast-food outlets | 0.00 | -0.05 | 0.05 | 0.98 |
| Interaction Factor 2 & Fast-food outlets | -0.05 | -0.11 | 0.00 | 0.07 |
|  |  |  |  |  |
| BMI | β | 95% LCI | 95% UCI | P-value |
| Factor 2 | -0.04 | -0.49 | 0.41 | 0.85 |
| Restaurants | 0.00 | -0.02 | 0.01 | 0.47 |
| Interaction Factor 2 & Restaurants | -0.01 | -0.02 | 0.00 | 0.09 |
|  |  |  |  |  |
| BMI | β | 95% LCI | 95% UCI | P-value |
| Factor 2 | -0.27 | -0.64 | 0.11 | 0.16 |
| Supermarkets | -0.18 | -0.42 | 0.05 | 0.13 |
| Interaction Factor 2 & Supermarkets | -0.06 | -0.31 | 0.20 | 0.67 |
|  |  |  |  |  |
| BMI | β | 95% LCI | 95% UCI | P-value |
| Factor 2 | -0.19 | -0.57 | 0.18 | 0.31 |
| Fruit and vegetable stores | -0.01 | -0.03 | 0.01 | 0.34 |
| Interaction Factor 2 & Fruit and vegetable stores | -0.02 | -0.04 | 0.00 | 0.10 |
|  |  |  |  |  |
| **Factor 3** |  |  |  |  |
| BMI | β | 95% LCI | 95% UCI | P-value |
| Factor 3 | -0.47 | -0.93 | -0.01 | 0.05 |
| Convenience stores | 0.00 | -0.01 | 0.01 | 0.96 |
| Interaction Factor 3 & Convenience stores | 0.00 | -0.01 | 0.01 | 0.68 |
|  |  |  |  |  |
| BMI | β | 95% LCI | 95% UCI | P-value |
| Factor 3 | -0.32 | -0.65 | 0.02 | 0.06 |
| Fast-food outlets | 0.03 | -0.01 | 0.07 | 0.15 |
| Interaction Factor 3 & Fast-food outlets | -0.01 | -0.05 | 0.02 | 0.39 |
|  |  |  |  |  |
| BMI | β | 95% LCI | 95% UCI | P-value |
| Factor 3 | -0.36 | -0.70 | -0.01 | 0.05 |
| Restaurants | 0.00 | -0.01 | 0.01 | 0.56 |
| Interaction Factor 3 & Restaurants | 0.00 | -0.01 | 0.01 | 0.71 |
|  |  |  |  |  |
| BMI | β | 95% LCI | 95% UCI | P-value |
| Factor 3 | -0.36 | -0.65 | -0.06 | **0.02** |
| Supermarkets | -0.15 | -0.38 | 0.07 | 0.18 |
| Interaction Factor 3 & Supermarkets | -0.06 | -0.26 | 0.13 | 0.53 |
|  |  |  |  |  |
| BMI | β | 95% LCI | 95% UCI | P-value |
| Factor 3 | -0.40 | -0.69 | -0.11 | **0.01** |
| Fruit and vegetable stores | 0.00 | -0.01 | 0.02 | 0.74 |
| Interaction Factor 3 & Fruit and vegetable stores | 0.00 | -0.01 | 0.01 | 0.84 |
|  |  |  |  |  |
| **MODEL B** |  |  |  |  |
| **Factor 1** |  |  |  |  |
| BMI | β | 95% LCI | 95% UCI | P-value |
| Factor 1 | -0.09 | -0.56 | 0.37 | 0.69 |
| Convenience stores | 0.00 | -0.01 | 0.01 | 0.85 |
| Interaction Factor 1 & Convenience stores | 0.00 | -0.01 | 0.01 | 0.66 |
|  |  |  |  |  |
| BMI | β | 95% LCI | 95% UCI | P-value |
| Factor 1 | -0.06 | -0.42 | 0.29 | 0.72 |
| Fast-food outlets | 0.04 | -0.01 | 0.08 | 0.11 |
| Interaction Factor 1 & Fast-food outlets | -0.03 | -0.07 | 0.02 | 0.28 |
|  |  |  |  |  |
| BMI | β | 95% LCI | 95% UCI | P-value |
| Factor 1 | -0.13 | -0.50 | 0.23 | 0.48 |
| Restaurants | 0.00 | -0.01 | 0.01 | 0.86 |
| Interaction Factor 1 & Restaurants | 0.00 | -0.01 | 0.01 | 0.70 |
|  |  |  |  |  |
| BMI | β | 95% LCI | 95% UCI | P-value |
| Factor 1 | -0.12 | -0.42 | 0.18 | 0.44 |
| Supermarkets | -0.13 | -0.37 | 0.10 | 0.27 |
| Interaction Factor 1 & Supermarkets | -0.12 | -0.33 | 0.09 | 0.26 |
|  |  |  |  |  |
| BMI | β | 95% LCI | 95% UCI | P-value |
| Factor 1 | -0.12 | -0.42 | 0.19 | 0.45 |
| Fruit and vegetable stores | 0.01 | -0.01 | 0.03 | 0.35 |
| Interaction Factor 1 & Fruit and vegetable stores | -0.01 | -0.03 | 0.01 | 0.18 |
|  |  |  |  |  |
| **Factor 2** |  |  |  |  |
| BMI | β | 95% LCI | 95% UCI | P-value |
| Factor 2 | 0.12 | -0.46 | 0.70 | 0.68 |
| Convenience stores | 0.00 | -0.01 | 0.00 | 0.35 |
| Interaction Factor 2 & Convenience stores | -0.01 | -0.02 | 0.00 | 0.13 |
|  |  |  |  |  |
| BMI | β | 95% LCI | 95% UCI | P-value |
| Factor 2 | 0.02 | -0.43 | 0.48 | 0.92 |
| Fast-food outlets | 0.01 | -0.04 | 0.05 | 0.79 |
| Interaction Factor 2 & Fast-food outlets | -0.05 | -0.10 | 0.01 | 0.09 |
|  |  |  |  |  |
| BMI | β | 95% LCI | 95% UCI | P-value |
| Factor 2 | 0.00 | -0.45 | 0.44 | 0.99 |
| Restaurants | -0.01 | -0.02 | 0.01 | 0.37 |
| Interaction Factor 2 & Restaurants | -0.01 | -0.02 | 0.00 | 0.09 |
|  |  |  |  |  |
| BMI | β | 95% LCI | 95% UCI | P-value |
| Factor 2 | -0.22 | -0.59 | 0.15 | 0.25 |
| Supermarkets | -0.19 | -0.43 | 0.05 | 0.12 |
| Interaction Factor 2 & Supermarkets | -0.05 | -0.30 | 0.21 | 0.71 |
|  |  |  |  |  |
| BMI | β | 95% LCI | 95% UCI | P-value |
| Factor 2 | -0.15 | -0.52 | 0.22 | 0.42 |
| Fruit and vegetable stores | -0.01 | -0.03 | 0.01 | 0.28 |
| Interaction Factor 2 & Fruit and vegetable stores | -0.02 | -0.04 | 0.00 | 0.11 |
|  |  |  |  |  |
| **Factor 3** |  |  |  |  |
| BMI | β | 95% LCI | 95% UCI | P-value |
| Factor 3 | -0.42 | -0.89 | 0.04 | 0.08 |
| Convenience stores | 0.00 | -0.01 | 0.01 | 0.73 |
| Interaction Factor 3 & Convenience stores | 0.00 | -0.01 | 0.01 | 0.80 |
|  |  |  |  |  |
| BMI | β | 95% LCI | 95% UCI | P-value |
| Factor 3 | -0.30 | -0.64 | 0.03 | 0.08 |
| Fast-food outlets | 0.03 | -0.01 | 0.08 | 0.11 |
| Interaction Factor 3 & Fast-food outlets | -0.02 | -0.05 | 0.02 | 0.37 |
|  |  |  |  |  |
| BMI | β | 95% LCI | 95% UCI | P-value |
| Factor 3 | -0.34 | -0.69 | 0.01 | 0.06 |
| Restaurants | 0.00 | -0.01 | 0.01 | 0.69 |
| Interaction Factor 3 & Restaurants | 0.00 | -0.01 | 0.01 | 0.70 |
|  |  |  |  |  |
| BMI | β | 95% LCI | 95% UCI | P-value |
| Factor 3 | -0.33 | -0.63 | -0.03 | **0.03** |
| Supermarkets | -0.17 | -0.39 | 0.06 | 0.15 |
| Interaction Factor 3 & Supermarkets | -0.08 | -0.28 | 0.11 | 0.41 |
|  |  |  |  |  |
| BMI | β | 95% LCI | 95% UCI | P-value |
| Factor 3 | -0.37 | -0.67 | -0.08 | **0.01** |
| Fruit and vegetable stores | 0.00 | -0.01 | 0.02 | 0.86 |
| Interaction Factor 3 & Fruit and vegetable stores | 0.00 | -0.01 | 0.01 | 0.98 |
|  |  |  |  |  |
| **MODEL C** |  |  |  |  |
| **Factor 1** |  |  |  |  |
| BMI | β | 95% LCI | 95% UCI | P-value |
| Factor 1 | -0.14 | -0.60 | 0.32 | 0.56 |
| Convenience stores | 0.00 | -0.01 | 0.01 | 0.95 |
| Interaction Factor 1 & Convenience stores | 0.00 | -0.01 | 0.01 | 0.63 |
|  |  |  |  |  |
| BMI | β | 95% LCI | 95% UCI | P-value |
| Factor 1 | -0.14 | -0.49 | 0.22 | 0.45 |
| Fast-food outlets | 0.03 | -0.01 | 0.08 | 0.15 |
| Interaction Factor 1 & Fast-food outlets | -0.02 | -0.07 | 0.03 | 0.40 |
|  |  |  |  |  |
| BMI | β | 95% LCI | 95% UCI | P-value |
| Factor 1 | -0.20 | -0.56 | 0.16 | 0.28 |
| Restaurants | 0.00 | -0.01 | 0.01 | 0.71 |
| Interaction Factor 1 & Restaurants | 0.00 | -0.01 | 0.01 | 0.81 |
|  |  |  |  |  |
| BMI | β | 95% LCI | 95% UCI | P-value |
| Factor 1 | -0.18 | -0.48 | 0.12 | 0.25 |
| Supermarkets | -0.12 | -0.36 | 0.11 | 0.30 |
| Interaction Factor 1 & Supermarkets | -0.10 | -0.31 | 0.11 | 0.34 |
|  |  |  |  |  |
| BMI | β | 95% LCI | 95% UCI | P-value |
| Factor 1 | -0.16 | -0.46 | 0.14 | 0.30 |
| Fruit and vegetable stores | 0.01 | -0.01 | 0.03 | 0.22 |
| Interaction Factor 1 & Fruit and vegetable stores | -0.01 | -0.03 | 0.00 | 0.12 |
|  |  |  |  |  |
| **Factor 2** |  |  |  |  |
| BMI | β | 95% LCI | 95% UCI | P-value |
| Factor 2 | 0.11 | -0.47 | 0.69 | 0.72 |
| Convenience stores | 0.00 | -0.01 | 0.01 | 0.51 |
| Interaction Factor 2 & Convenience stores | -0.01 | -0.02 | 0.00 | 0.10 |
|  |  |  |  |  |
| BMI | β | 95% LCI | 95% UCI | P-value |
| Factor 2 | 0.00 | -0.46 | 0.46 | 1.00 |
| Fast-food outlets | 0.00 | -0.05 | 0.05 | 0.94 |
| Interaction Factor 2 & Fast-food outlets | -0.05 | -0.11 | 0.00 | 0.07 |
|  |  |  |  | 0.12 |
| BMI | β | 95% LCI | 95% UCI | P-value |
| Factor 2 | -0.04 | -0.49 | 0.41 | 0.86 |
| Restaurants | 0.00 | -0.02 | 0.01 | 0.48 |
| Interaction Factor 2 & Restaurants | -0.01 | -0.02 | 0.00 | 0.09 |
|  |  |  |  |  |
| BMI | β | 95% LCI | 95% UCI | P-value |
| Factor 2 | -0.27 | -0.64 | 0.11 | 0.16 |
| Supermarkets | -0.18 | -0.42 | 0.05 | 0.13 |
| Interaction Factor 2 & Supermarkets | -0.06 | -0.31 | 0.20 | 0.67 |
|  |  |  |  |  |
| BMI | β | 95% LCI | 95% UCI | P-value |
| Factor 2 | -0.19 | -0.56 | 0.18 | 0.31 |
| Fruit and vegetable stores | -0.01 | -0.03 | 0.01 | 0.33 |
| Interaction Factor 1 & Fruit and vegetable stores | -0.02 | -0.04 | 0.00 | 0.09 |
|  |  |  |  |  |
| **Factor 3** |  |  |  |  |
| BMI | β | 95% LCI | 95% UCI | P-value |
| Factor 3 | -0.46 | -0.93 | 0.00 | 0.05 |
| Convenience stores | 0.00 | -0.01 | 0.01 | 0.93 |
| Interaction Factor 3 & Convenience stores | 0.00 | -0.01 | 0.01 | 0.68 |
|  |  |  |  |  |
| BMI | β | 95% LCI | 95% UCI | P-value |
| Factor 3 | -0.31 | -0.64 | 0.02 | 0.07 |
| Fast-food outlets | 0.03 | -0.01 | 0.07 | 0.14 |
| Interaction Factor 3 & Fast-food outlets | -0.02 | -0.05 | 0.02 | 0.37 |
|  |  |  |  |  |
| BMI | β | 95% LCI | 95% UCI | P-value |
| Factor 3 | -0.35 | -0.70 | 0.00 | 0.05 |
| Restaurants | 0.00 | -0.01 | 0.01 | 0.56 |
| Interaction Factor 3 & Restaurants | 0.00 | -0.01 | 0.01 | 0.70 |
|  |  |  |  |  |
| BMI | β | 95% LCI | 95% UCI | P-value |
| Factor 3 | -0.35 | -0.65 | -0.06 | **0.02** |
| Supermarkets | -0.15 | -0.38 | 0.07 | 0.18 |
| Interaction Factor 3 & Supermarkets | -0.06 | -0.26 | 0.13 | 0.53 |
|  |  |  |  |  |
| BMI | β | 95% LCI | 95% UCI | P-value |
| Factor 3 | -0.40 | -0.69 | -0.10 | **0.01** |
| Fruit and vegetable stores | 0.00 | -0.01 | 0.02 | 0.74 |
| Interaction Factor 3 & Fruit and vegetable stores | 0.00 | -0.01 | 0.01 | 0.84 |

BMI: body mass index. LCI: lower confidence interval. UCI: upper confidence interval

Model A: Age, sex, and socioeconomic position, N = 1,572

Model B: Model A + socioeconomic position, physical activity, car ownership, neighbourhood deprivation level, CTA (2nd level), N = 1,568

Model C: Model A + deprivation and urbanity of CTA, N = 1,572

Results indicate β coefficients and 95% confidence intervals

Bold values indicate statistically significant values (P < 0.05)
