# Supplementary material for: Association of the retail food environment, BMI, dietary patterns, and socioeconomic position in urban areas of Mexico
Source: PLOS Glob Public Health. 2023 Feb 23;3(2):e0001069. doi: 10.1371/journal.pgph.0001069 (PMC10022358; doi:10.1371/journal.pgph.0001069)
Supplement: S3 Table — (DOCX) [file pgph.0001069.s005.docx]

**S3 Table. Association of the food environment and BMI whilst considering dietary patterns as a confounder**

| **Model A** |  |  |  |  |
| --- | --- | --- | --- | --- |
| **Factor 1** |  |  |  |  |
| **BMI** | **β** | **95% LCI** | **95% UCI** | **P-value** |
| Convenience stores | 0.00 | -0.01 | 0.01 | 0.93 |
| Fast-food outlets | 0.03 | -0.02 | 0.07 | 0.23 |
| Restaurants | 0.00 | -0.01 | 0.01 | 0.77 |
| Supermarkets | -0.15 | -0.38 | 0.07 | 0.18 |
| Fruit and vegetable stores | 0.00 | -0.01 | 0.02 | 0.71 |
| **Factor 2** |  |  |  |  |
| Convenience stores | 0.03 | -0.03 | 0.09 | 0.30 |
| Fast-food outlets | 0.02 | -0.02 | 0.07 | 0.26 |
| Restaurants | 0.00 | -0.01 | 0.01 | 0.87 |
| Supermarkets | -0.17 | -0.39 | 0.06 | 0.15 |
| Fruit and vegetable stores | 0.00 | -0.01 | 0.02 | 0.84 |
| **Factor 3** |  |  |  |  |
| Convenience stores | 0.00 | -0.01 | 0.01 | 0.86 |
| Fast-food outlets | 0.03 | -0.01 | 0.07 | 0.19 |
| Restaurants | 0.00 | -0.01 | 0.01 | 0.61 |
| Supermarkets | -0.15 | -0.38 | 0.07 | 0.18 |
| Fruit and vegetable stores | 0.00 | -0.01 | 0.02 | 0.73 |
| **MODEL B** |  |  |  |  |
| **Factor 1** |  |  |  |  |
| Convenience stores | 0.00 | -0.01 | 0.01 | 0.68 |
| Fast-food outlets | 0.03 | -0.01 | 0.07 | 0.18 |
| Restaurants | 0.00 | -0.01 | 0.01 | 0.95 |
| Supermarkets | -0.17 | -0.40 | 0.06 | 0.14 |
| Fruit and vegetable stores | 0.00 | -0.01 | 0.02 | 0.89 |
| **Factor 2** |  |  |  |  |
| Convenience stores | 0.06 | 0.01 | 0.12 | **0.03** |
| Fast-food outlets | 0.03 | -0.01 | 0.07 | 0.20 |
| Restaurants | 0.00 | -0.01 | 0.01 | 0.94 |
| Supermarkets | -0.18 | -0.40 | 0.05 | 0.13 |
| Fruit and vegetable stores | 0.00 | -0.01 | 0.01 | 0.96 |
| **Factor 3** |  |  |  |  |
| Convenience stores | 0.00 | -0.01 | 0.01 | 0.66 |
| Fast-food outlets | 0.03 | -0.01 | 0.07 | 0.14 |
| Restaurants | 0.00 | -0.01 | 0.01 | 0.75 |
| Supermarkets | -0.17 | -0.39 | 0.06 | 0.15 |
| Fruit and vegetable stores | 0.00 | -0.01 | 0.02 | 0.86 |
| ***MODEL C** |  |  |  |  |
| **Factor 1** |  |  |  |  |
| Convenience stores | 0.00 | -0.01 | 0.01 | 0.89 |
| Fast-food outlets | 0.03 | -0.02 | 0.07 | 0.21 |
| Restaurants | 0.00 | -0.01 | 0.01 | 0.75 |
| Supermarkets | -0.16 | -0.38 | 0.07 | 0.18 |
| Fruit and vegetable stores | 0.00 | -0.01 | 0.02 | 0.72 |
| **Factor 2** |  |  |  |  |
| Convenience stores | 0.03 | -0.03 | 0.09 | 0.29 |
| Fast-food outlets | 0.03 | -0.02 | 0.07 | 0.24 |
| Restaurants | 0.00 | -0.01 | 0.01 | 0.86 |
| Supermarkets | -0.17 | -0.39 | 0.06 | 0.15 |
| Fruit and vegetable stores | 0.00 | -0.01 | 0.02 | 0.85 |
| **Factor 3** |  |  |  |  |
| Convenience stores | 0.00 | -0.01 | 0.01 | 0.84 |
| Fast-food outlets | 0.03 | -0.01 | 0.07 | 0.18 |
| Restaurants | 0.00 | -0.01 | 0.01 | 0.61 |
| Supermarkets | -0.16 | -0.38 | 0.07 | 0.18 |
| Fruit and vegetable stores | 0.00 | -0.01 | 0.02 | 0.74 |

BMI: body mass index. LCI: lower confidence interval. UCI: upper confidence interval

Model A: Age, sex, and socioeconomic position, N = 1,572

Model B: Model A + socioeconomic position, physical activity, car ownership, neighbourhood deprivation level, CTA (2nd level), N = 1,568

Model C: Model A + deprivation and urbanity of CTA, N = 1,572

Results indicate β coefficients and 95% confidence intervals

Bold values indicate statistically significant values (P < 0.05)
