# Supplementary material for: Association of the retail food environment, BMI, dietary patterns, and socioeconomic position in urban areas of Mexico
Source: PLOS Glob Public Health. 2023 Feb 23;3(2):e0001069. doi: 10.1371/journal.pgph.0001069 (PMC10022358; doi:10.1371/journal.pgph.0001069)
Supplement: S4 Table — (DOCX) [file pgph.0001069.s006.docx]

**S4 Table. Association of the food environment and obesity accounting for dietary patterns and stratifying by sex***

|  | **Male** | | | | **Female** | | | |
| --- | --- | --- | --- | --- | --- | --- | --- | --- |
| **BMI** | **β** | **95% UCI** | **95% LCI** | **P-value** | **β** | **95% UCI** | **95% LCI** | **P-value** |
| **Factor 1** |  |  |  |  |  |  |  |  |
| Convenience stores | 0.00 | -0.01 | 0.01 | 0.61 | -0.01 | -0.07 | 0.06 | 0.86 |
| Fast-food outlet | 0.00 | -0.07 | 0.06 | 0.90 | 0.04 | -0.01 | 0.09 | 0.15 |
| Restaurant | -0.01 | -0.02 | 0.01 | 0.34 | 0.01 | -0.01 | 0.02 | 0.37 |
| Supermarket | -0.02 | -0.36 | 0.31 | 0.90 | -0.20 | -0.50 | 0.10 | 0.20 |
| Fruit and vegetable stores | -0.01 | -0.03 | 0.02 | 0.64 | 0.01 | -0.01 | 0.02 | 0.53 |
| **Factor 2** |  |  |  |  |  |  |  |  |
| Convenience stores | -0.01 | -0.02 | 0.01 | 0.28 | 0.00 | -0.01 | 0.01 | 0.64 |
| Fast-food outlet | -0.01 | -0.07 | 0.06 | 0.86 | 0.04 | -0.01 | 0.09 | 0.16 |
| Restaurant | -0.01 | -0.02 | 0.01 | 0.29 | 0.01 | -0.01 | 0.02 | 0.41 |
| Supermarket | -0.05 | -0.38 | 0.29 | 0.79 | -0.20 | -0.50 | 0.10 | 0.18 |
| Fruit and vegetable stores | -0.01 | -0.03 | 0.02 | 0.58 | 0.00 | -0.01 | 0.02 | 0.58 |
| **Factor 3** |  |  |  |  |  |  |  |  |
| Convenience stores | -0.01 | -0.02 | 0.01 | 0.31 | 0.00 | -0.01 | 0.01 | 0.76 |
| Fast-food outlet | 0.00 | -0.07 | 0.06 | 0.89 | 0.04 | -0.01 | 0.10 | 0.12 |
| Restaurant | -0.01 | -0.02 | 0.01 | 0.33 | 0.01 | -0.01 | 0.02 | 0.29 |
| Supermarket | -0.03 | -0.37 | 0.30 | 0.85 | -0.20 | -0.50 | 0.10 | 0.19 |
| Fruit and vegetable stores | -0.01 | -0.03 | 0.02 | 0.65 | 0.00 | -0.01 | 0.02 | 0.56 |

*The association was tested considering Model A and adjusting for sociodemographic characteristics.

Model A: Age, sex, and socioeconomic position, N = 1,572

BMI: body mass index. LCI: lower confidence interval. UCI: upper confidence interval.

Results indicate β coefficients and 95% confidence intervals
